# A New Troodontid Theropod, *Talos sampsoni* gen. et sp. nov., from the Upper Cretaceous Western Interior Basin of North America

**DOI:** 10.1371/journal.pone.0024487

**Published:** 2011-09-19

**Authors:** Lindsay E. Zanno, David J. Varricchio, Patrick M. O'Connor, Alan L. Titus, Michael J. Knell

**Affiliations:** 1 Field Museum of Natural History, Chicago, Illinois, United States of America; 2 Biological Sciences Department, University of Wisconsin-Parkside, Kenosha, Wisconsin, United States of America; 3 Department of Earth Sciences, Montana State University, Bozeman, Montana, United States of America; 4 Department of Biomedical Sciences, Ohio University College of Osteopathic Medicine, Athens, Ohio, United States of America; 5 Ohio Center for Ecology and Evolutionary Studies, Ohio University, Athens, Ohio, United States of America; 6 Grand Staircase-Escalante National Monument, Bureau of Land Management, Kanab, Utah, United States of America; Institut de Biologia Evolutiva - Universitat Pompeu Fabra, Spain

## Abstract

**Background:**

Troodontids are a predominantly small-bodied group of feathered theropod dinosaurs notable for their close evolutionary relationship with Avialae. Despite a diverse Asian representation with remarkable growth in recent years, the North American record of the clade remains poor, with only one controversial species—*Troodon formosus*—presently known from substantial skeletal remains.

**Methodology/Principal Findings:**

Here we report a gracile new troodontid theropod—*Talos sampsoni* gen. et sp. nov.—from the Upper Cretaceous Kaiparowits Formation, Utah, USA, representing one of the most complete troodontid skeletons described from North America to date. Histological assessment of the holotype specimen indicates that the adult body size of *Talos* was notably smaller than that of the contemporary genus *Troodon*. Phylogenetic analysis recovers *Talos* as a member of a derived, latest Cretaceous subclade, minimally containing *Troodon*, *Saurornithoides*, and *Zanabazar*. MicroCT scans reveal extreme pathological remodeling on pedal phalanx II-1 of the holotype specimen likely resulting from physical trauma and subsequent infectious processes.

**Conclusion/Significance:**

*Talos sampsoni* adds to the singularity of the Kaiparowits Formation dinosaur fauna, which is represented by at least 10 previously unrecognized species including the recently named ceratopsids *Utahceratops* and *Kosmoceratops*, the hadrosaurine *Gryposaurus monumentensis*, the tyrannosaurid *Teratophoneus*, and the oviraptorosaurian *Hagryphus*. The presence of a distinct troodontid taxon in the Kaiparowits Formation supports the hypothesis that late Campanian dinosaurs of the Western Interior Basin exhibited restricted geographic ranges and suggests that the taxonomic diversity of Late Cretaceous troodontids from North America is currently underestimated. An apparent traumatic injury to the foot of *Talos* with evidence of subsequent healing sheds new light on the paleobiology of deinonychosaurians by bolstering functional interpretations of prey grappling and/or intraspecific combat for the second pedal digit, and supporting trackway evidence indicating a minimal role in weight bearing.

## Introduction

Troodontids are an intriguing clade of feathered theropods known to possess the smallest body sizes [1]–[3] and largest relative brain sizes [4] among non-avian dinosaurs, and widely hypothesized to be among the closest extinct relatives of birds [2], [5]–[7]. Until recently, the fossil record of troodontids was poorly known. However, the past decade has witnessed the discovery of several remarkable specimens that not only support a close relationship with Avialae but also provide unprecedented glimpses into the biology of non-avian dinosaurs. These include exemplary specimens documenting transitional phases in the evolution of avian reproductive physiology and behavior [8]–[11], as well as those displaying evidence of an avian-style sleeping posture [12]–[13], ‘four-winged’ locomotor capabilities [3], and perhaps most extraordinary, plumage coloration [14].

Recent discoveries notwithstanding, troodontids are among the rarest of dinosaurs. To date, the taxonomic diversity of the clade is confined almost exclusively to Asia, where exceptional preservation permits the documentation of at least 14 species. In contrast, the taxonomic resolution of North American troodontid materials is obfuscated by a paucity of well-preserved skulls and associated skeletons. This preservational bias has resulted in a series of reclassifications and synonymizations that span over 150 years of scientific inquiry and remain a continuous point of debate. Currently the diversity of North American troodontids is restricted to three or more recognized Cretaceous forms including *Troodon formosus* [15]–[16], *Troodon inequalis* [4], *Geminiraptor suarezarum* [17], and the tooth taxon *Pectinodon bakkeri* [18]–[19], as well as two putative Late Jurassic taxa, including the tooth taxon *Koparion douglassi* [20] and a possible new species from the Morrison Formation [21].

Here we report the discovery of a new troodontid theropod—*Talos sampsoni*—from the Upper Cretaceous Kaiparowits Formation, Grand Staircase-Escalante National Monument, southern Utah, USA. Although an isolated tooth taxon (*Pectinodon*) [18] was referred to Troodontidae since the coining of *Stenonychosaurus inequalis* in 1932 [22], *Talos* represents the first definitive North American troodontid from the Late Cretaceous to be named in over 75 years and sheds new light on the taxonomic and paleobiogeographic diversity of North American dinosaurs of the Western Interior Basin.

### Institutional Abbreviations

**AMNH**: American Museum of Natural History, New York, New York, USA; **IGM**: Geological Institute of the Mongolian Academy of Sciences, Ulaan Bataar, Mongolia; **IVPP**: Institute of Vertebrate Palaeontology and Palaeoanthropology, Chinese Academy of Sciences, Beijing, China; **CMN**: Canadian Museum of Nature, Ottawa, Ontario, Canada; **MOR**: Museum of the Rockies, Bozeman, Montana, USA; **TMP**: Royal Tyrrell Museum of Palaeontology, Drumheller, Alberta, Canada; **UMNH**: Utah Museum of Natural History, Salt Lake City, Utah, USA.

## Materials and Methods

### Taxonomic Considerations

Historically, Late Cretaceous North American troodontid materials were classified under the nomina *Troodon formosus* [23], *Polyodontosaurus grandis* [24], and *Stenonychosaurus inequalis* [22]. Since their establishment, these names have been the subject of continuous instability. Romer [25] synonymized *Polyodontosaurus* (an isolated jaw) with *Troodon formosus*. Soon thereafter Russell [26] synonymized *Polyodontosaurus,* and *Ornithomimus altus* [27] under *Stenonychosaurus inequalis* based on new materials of the latter, yet noted a lack of sufficient evidence to synonymize these materials with *Troodon formosus*. Five years later, Barsbold [28] named a second species of *Saurornithoides* (*S. junior*), upheld the synonymizations of Russell [26], further noted the distinctiveness of *Troodon* and *Stenonychosaurus*, and erected a new higher-level taxon (Saurornithoididae) for *Saurornithoides* and *Stenonychosaurus* to the exclusion of *T*. *formosus*. Carpenter [18] subsequently coined a new name *Pectinodon bakkeri* for a sample of troodontid teeth from the Maastrichtian Lance Formation and synonymized *Polyodontosaurus*, *Stenonychosaurus*, *O*. *altus*, and *T*. *formosus* under the new combination *Saurornithoides inequalis*, to which he referred a partial dentary and basioccipital.

Most recently the genus *Troodon* has taken precedence over *Stenonychosaurus* in the literature. Based on the discovery of a troodontid dentary (TMP 83.12.11) from the Horseshoe Canyon Formation and a review of North American troodontid materials, Currie [15] synonymized *Stenonychosaurus inequalis* and *Pectinodon bakkeri* under *Troodon formosus*. Thereafter, the name *Troodon formosus* was applied to nearly all troodontid materials recovered from the Upper Cretaceous of North America [16], [29]–[32]. More recently Currie [4] proposed *Troodon inequalis* as a more restricted designation for troodontid materials recovered from Dinosaur Provincial Park.

Given the contention regarding the taxonomy of Late Cretaceous North American troodontid materials and the status of *Troodon formosus* in particular (see 4, 15, 18–19, 26, 33], we recognize the possibility that specimens currently constituting the hypodigm “*Troodon formosus*” from these and other formations of the Western Interior Basin may represent multiple species and that *Troodon formosus*, since based on a single tooth [23], may represent a *nomen dubium*. In order to maintain taxonomic stability until such time as *T*. *formosus* is reevaluated and for the purpose of morphological comparison, we herein follow Currie [15], Varricchio et al. [8]–[9], Varricchio and Jackson [10], and Makovicky and Norell [16] in regarding troodontid specimens from the Oldman, Judith River, Two Medicine, and Dinosaur Park formations as *Troodon formosus*. Nevertheless, expecting that some of these specimens may be reclassified in the near future, we refer explicitly to specimen numbers when we make comparative statements between *Talos sampsoni* and *Troodon formosus*.

### Histology

Petrographic specimens of UMNH VP 19479 were prepared at the Field Museum of Natural History (Chicago, IL). Transverse sections were obtained from the proximal shaft of the femur and fibula, and midshaft of the fibula to minimize damage to the holotype specimen. Casts of pre-sectioned elements and petrographic sections are reposited at the UMNH. Sections were embedded in Castalite-AP® acrylic-polyester resin, sectioned using a diamond blade, polished successively using 600 and 1,200 grit silicon carbide grinding paper, and micropolished with a de-agglomerated alpha alumina powder (0.3 µm), water, and soap solution.

### μCT Scanning

To assess the degree of pathological modification on UMNH VP 19479, select elements of the left and right ankle and pes were scanned at the Ohio University microCT (OUμCT) Facility (http://www.oucom.ohiou.edu/ou-microct/) on a GE eXplore Locus *in-vivo* micro-CT scanner. Scan data were acquired at 85 kVp, 400 mA, and a slice thickness of 0.045 mm, with digital post-processing completed in GE MicroView 2.1.2 and AMIRA 4.1 Advanced Graphics Package.

### Phylogenetic Protocol

To investigate its evolutionary relationships, we added *Talos sampsoni* and the primitive troodontids *Mei long* [13] and *Sinovenator changi* [5] to the character matrix of Norell et al. [34]. Character coding, manipulation, and tree visualization were carried out using Mesquite ver. 2.74 [35]. Analyses were executed in the program TNT (Trees using New Technology), provided by the Willi Hennig Society [36]. Most parsimonious trees (MPTs) were obtained via heuristic search methods on 1000 replicates of Wagner trees with random addition sequences and then subject to TBR (tree bisection-reconnection) swapping methods holding 10 trees per replicate. Results were assessed using strict and reduced consensus methods. Ambiguous nodes were collapsed following Rule 1 of Coddington & Sharff [37]. Maximum agreement subtrees [38] were used to identify labile taxa and common topology among all MPTs (most parisimonious trees). Standard bootstrapping (1000 replicates, GC values) [39], symmetrical resampling (1000 replicates, GC values) [40], and Bremer support were conducted to assess node stability. We retained the operational taxonomic units (OTUs) Dromaeosauridae and Avialae yet eliminated the extraneous and generalized OTUs Ornithomimidae and Oviraptorosauridae included in the original Norell et al. study [34] since our intent was not to examine outgroup relationships of Troodontidae and higher-level OTUs generally contain a mosaic of character states. We supplemented missing codes for several characters based on recent literature (2, 9, 34), modified one character (32) to capture ingroup rather than outgroup variation, and added 6 characters derived from our observation of *Talos* and specimens referred to *Troodon formosus*. The character list and matrix are available as Dataset S1.

### Nomenclatural Acts

The electronic version of this document does not represent a published work according to the International Code of Zoological Nomenclature (ICZN), and hence the nomenclatural acts contained in the electronic version are not available under that Code from the electronic edition. Therefore, a separate edition of this document was produced by a method that assures numerous identical and durable copies, and those copies were simultaneously obtainable (from the publication date noted on the first page of this article) for the purpose of providing a public and permanent scientific record, in accordance with Article 8.1 of the Code. The separate print-only edition is available on request from PLoS by sending a request to PLoS ONE, Public Library of Science, 1160 Battery Street, Suite 100, San Francisco, CA 94111, USA along with a check for $10 (to cover printing and postage) payable to “Public Library of Science”.

In addition, this published work and the nomenclatural acts it contains have been registered in ZooBank, the proposed online registration system for the ICZN. The ZooBank LSIDs (Life Science Identifiers) can be resolved and the associated information viewed through any standard web browser by appending the LSID to the prefix “http://zoobank.org/”. The LSID for this publication is (urn:lsid:zoobank.org:pub:9CC7B21F-2755-4EBD-AAD3-26EF1D6C3D7B).

### Fieldwork

All necessary permits were obtained for the described field studies. Fieldwork resulting in the recovery of UMNH VP 19479 was conducted with permission of Grand Staircase-Escalante National Monument and the Bureau of Land Management's National Landscape Conservation System (Assistance Agreement JSA071004).

## Results

### Systematic paleontology

Dinosauria Owen, 1842 [41].

Theropoda Marsh, 1881 [42].

Coelurosauria von Huene, 1914 [43].

Paraves Sereno, 1997 [44].

Troodontidae Gilmore, 1924 [45].

*Talos sampsoni* gen. et sp. nov.

### *Talos* gen. nov

ZooBank LSID urn∶lsid∶zoobank.org∶act∶963F60E2-4BCF-43EC-9C39-1BC81BE51AD8.

### *Talos sampsoni* sp. nov

ZooBank LSID urn∶lsid∶zoobank.org∶act∶08E4DCC9-80F3-4870-826F-1536BFD0A0BD.

**Figure 1:**
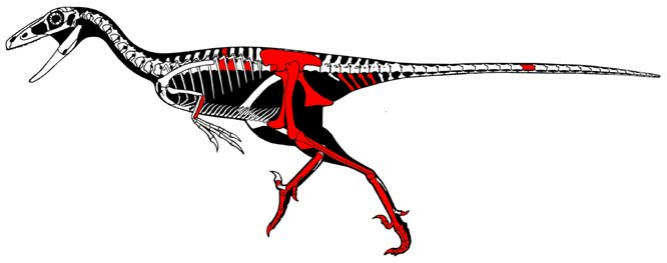
Skeletal reconstruction of *Talos sampsoni* (UMNH VP 19479). *Talos* is known from the upper Campanian Kaiparowits Formation, southern Utah, USA. Skeletal drawing illustrates the distribution of elements preserved with the holotype (in red). Missing elements based on *Troodon formosus* and/or *Saurornithoides mongoliensis*. Drawing © Scott Hartman 2010.

Figures 1, 3, 5–12.

#### Etymology

*Talos*, (Greek) referring to the mythological, fleet-footed protector of Crete, often depicted as winged, who succumbed to a wound on the ankle. The name is also a play on the English word “talon” meaning a sharply hooked claw. The specific epithet honors Scott D. Sampson, architect of the Kaiparowits Basin Project.

#### Holotype

UMNH VP 19479, partial postcranial skeleton, including fragmentary portions of the dorsal, sacral, and caudal axial column, left ulna, additional forelimb fragments, a partial pelvis, and partial left and right hind limbs (Figure 1).

#### Type Horizon, Locality, and Age

**Figure 2:**
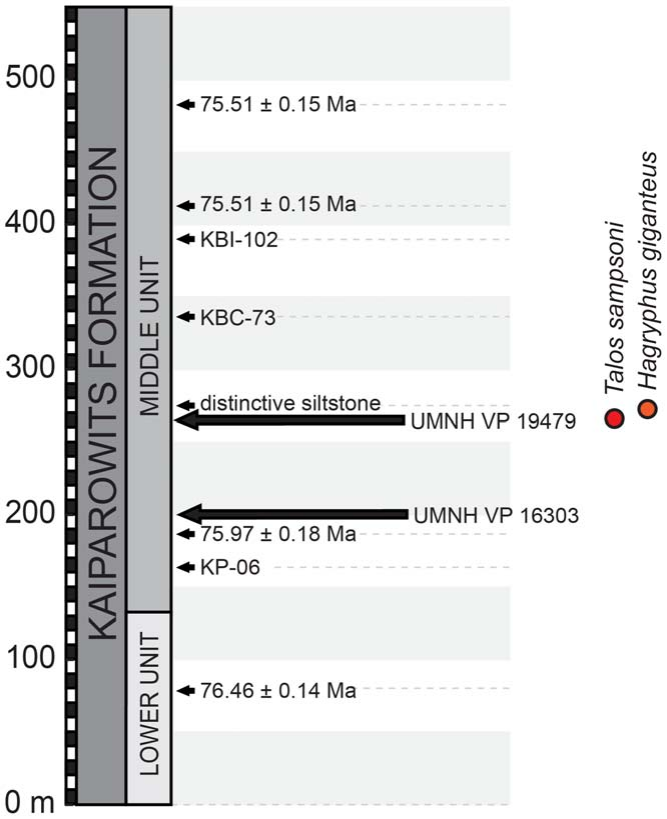
Stylized stratigraphic section of the middle and lower units of the Kaiparowits Formation [46]. Section indicates relative chronostratigraphic positions of *Talos sampsoni* UMNH VP 19479, troodontid indet. UMNH VP 16303, and *Hagryphus giganteus* (UMNH VP 12765). Radiometric dates derived from ^40^Ar/^39^Ar dating of bentonites [46], undated bentonite horizons also shown.

UMNH VP 19479 was collected in Grand Staircase-Escalante National Monument from a region known as “The Blues,” within the northern portion of the Kaiparowits Plateau. The specimen was entombed in a light greenish gray siltstone/fine sandstone unit exposed in the middle portion of the “middle unit” [46] of the Kaiparowits Formation, approximately 260 meters above the base (Figure 2). Small freshwater gastropod and bivalve shells (not collected) were observed associated with the skeleton and support the interpretation of the type horizon as overbank or possibly crevasse splay. A couple of small fragments of compactituberculate ornamented (sensu [47]) dinosaur eggshell were also observed at the locality. Small, 1–2 cm diameter calcium carbonate pedogenic nodules present at the bone level, as well as the blocky weathering nature of the host matrix, indicate that the horizon was a hypomature paleosol by the time it was buried deep enough to be isolated from soil forming processes. A bentonite located approximately 185 meters above the base of the Kaiparowits in this area (75 meters below the specimen; Figure 2) yielded a single crystal 40Ar/39Ar date of 75.95 +/− 0.18 Ma [48], indicating a late Campanian age for UMNH VP 19479.

**Figure 3:**
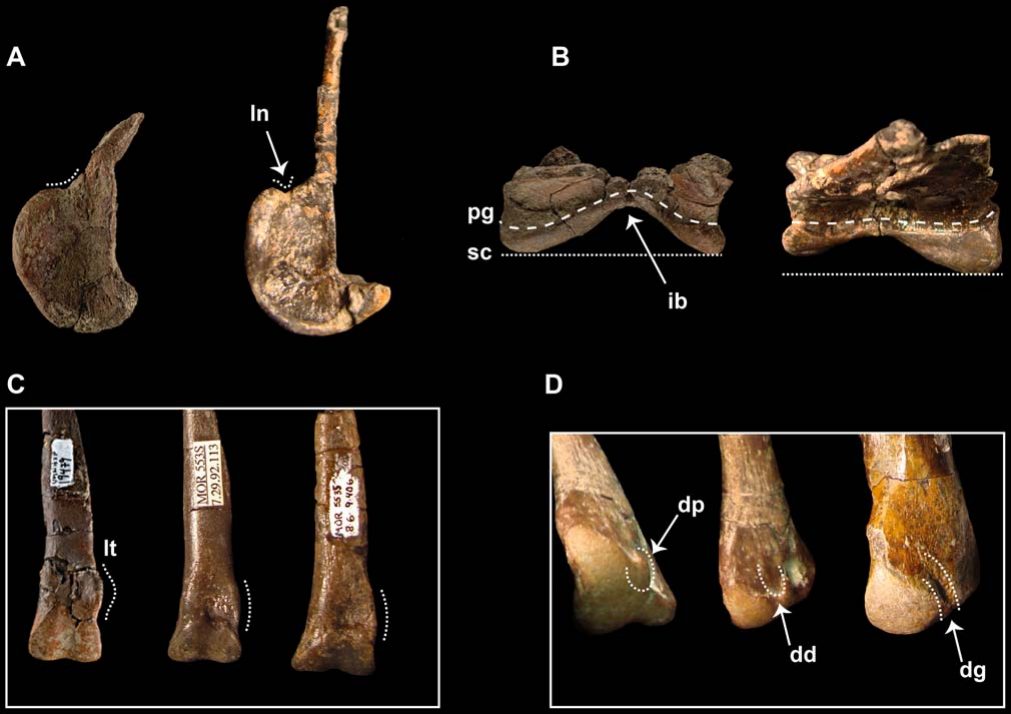
Skeletal elements of *Talos* and *Troodon* illustrating select diagnostic characters of *Talos sampsoni* (UMNH VP 19479). (**A**) left astragalus of *Talos* (on left) and right astragalus of *Troodon* (on right, reversed, MOR 553S-8.20.92.311) in lateral view, (**B**) and in proximal view, (**C**) distal aspect of left metatarsal III of *Talos* (on left) and *Troodon* (right two elements, MOR 553S-8.69.406, 7.29.92.113) in extensor view, and (**D**) distal aspect of left metatarsal IV of *Talos* (left two elements) and *Troodon* (on right, reversed, MOR 553S-11.1.01.8) in oblique distal and extensor, and extensor views respectively. Abbreviations: **dd**, dorsolateral depression on metatarsal IV; **dp**, dorsolateral tuberosity on metatarsal IV; **dg**, dorsolateral groove on metatarsal IV; **ib**, intercondylar bridge on astragalus; **ln**, lateral notch on astragalus; **lt**, lateral tab on metatarsal III; **pg**, proximal groove on astragalus; **sc**, subequal lateral and medial condyles of astragalus.

#### Diagnosis

**Figure 4:**
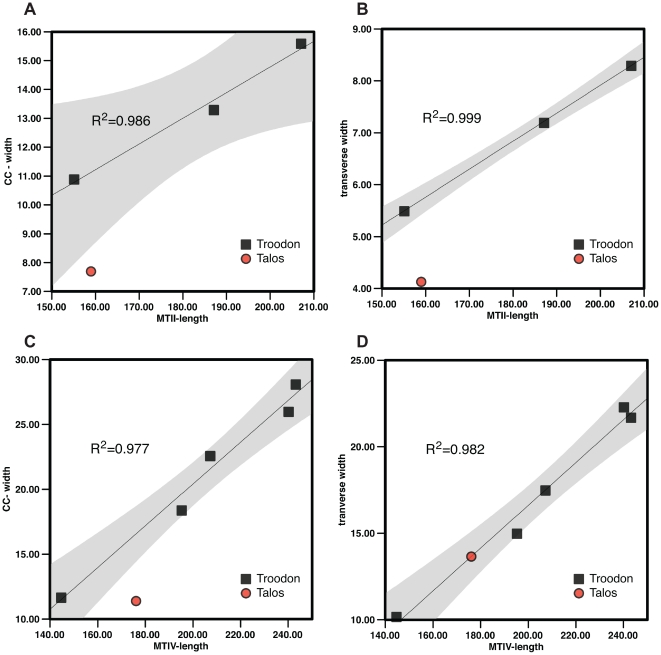
Linear regression of length-to-width ratio in the second and fourth metatarsals of *Talos sampsoni* and *Troodon formosus*. Regressions quantify the taxonomic significance of metatarsal slenderness in the holotype of *Talos* (UMNH VP 19479) based on transverse width of the second metatarsal (p<0.05) and craniocaudal width of the fourth metatarsal (p<0.05), despite its juvenile ontogenetic stage. *Troodon* specimens represented: metatarsal II (MOR 553S-7.8.91.28; MOR 553S-7.18.92.5; and MOR 748); metatarsal IV (MOR 553S-11.1.01.8; MOR 553S7.28.8.102; MOR 553S-7.1.9.9, MOR 553S-92.260, MOR 748).

A troodontid dinosaur exhibiting the following combination of characters (autapomorphies marked with asterisk): acetabular margin of ischium strongly concave dorsally; notch between lateral condyle and ascending process of astragalus in lateral view, poorly developed (Figure 3A); intercondylar bridge of astragalus hyperconstricted* (Figure 3B); proximal groove separating astragalar body and ascending process craniocaudally wide and v-shaped* (Figure 3B); cranioproximal groove on astragalar condyles absent; astragalar condyles subequal in cranial extent (Figure 3B); shaft of metatarsal II markedly compressed (midshaft length-to-transverse width ratio 38.8; Figure 4B); metatarsal III distally ginglymoid; pronounced, rounded tab on dorsolateral aspect of metatarsal III proximal to distal condyle (Figure 3C); distal corner of lateral collateral ligament pit on metatarsal IV with small protuberance, creating rounded depression between extensor aspects of distal condyles* (Figure 3D).

**Figure 5:**
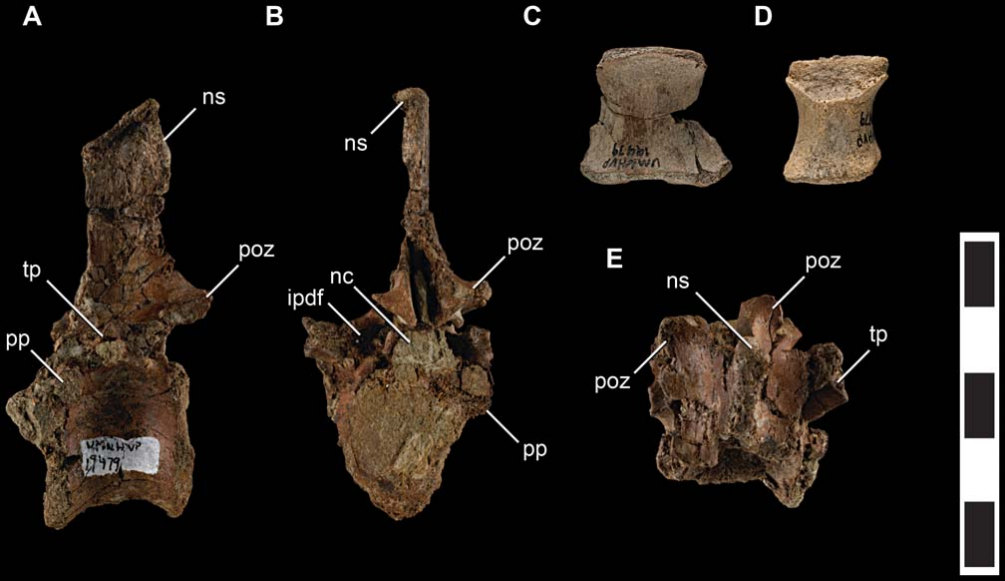
Axial elements of *Talos sampsoni* (UMNH VP 19479). Dorsal vertebra in left lateral (**A**), caudal (**B**), and dorsal (**E**) views; sacral centrum in ventral view (**C**), and dorsal centrum in ventral view (**D**). Abbreviations: **ipdf**, infrapostzygapophyseal fossa; **nc**, neural canal; **ns**, neural spine; **poz**, postzygapophysis; **pp**, parapophysis; **tp**, transverse process. Scale bar equals 5 cm.

**Figure 6 pone-0024487-g006:**
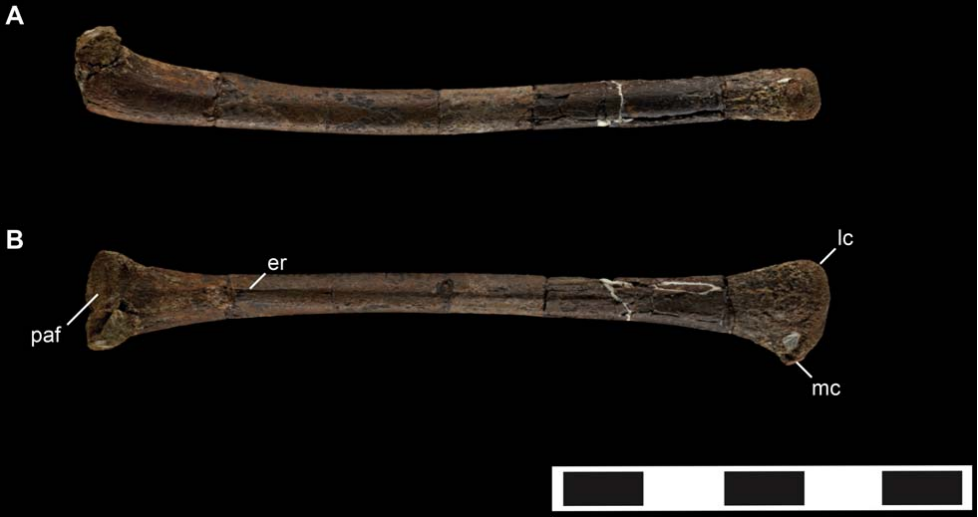
Left ulna of *Talos sampsoni* (UMNH VP 19479). Ulna in medial (**A**) and dorsal (**B**) views. Abbreviations: **er**, extensor ridge; **lc**, lateral aspect of the distal condyle condyle; **mc**, medial aspect of the distal condyle; **paf**, proximal articular facet. Scale bar equals 5 cm.

**Figure 7 pone-0024487-g007:**
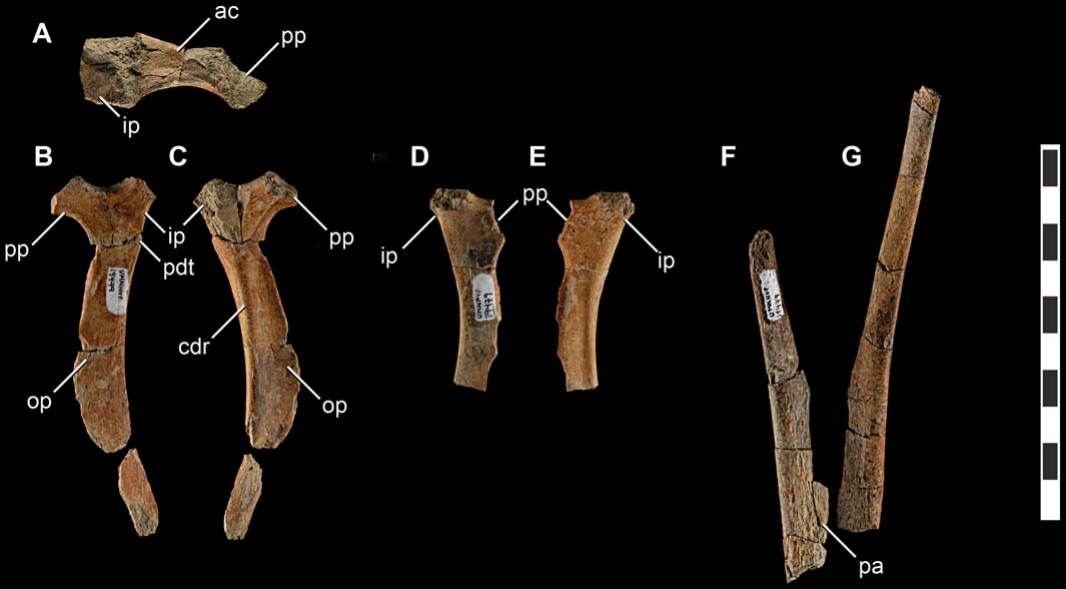
Pelvic girdle elements of *Talos sampsoni* (UMNH VP 19479). Right ischium in proximal (**A**), medial (**B**), and lateral (**C**) views; left ischium in medial (**D**) and lateral (**E**) views; right (**F**) and left (**G**) pubic shafts in cranial view. Abbreviations: **ac**, acetabulum; **cdr**, caudolateral ridge; **ip**, iliac peduncle; **op**, obturator process; **pa**, pubic apron; **pdt**, proximodorsal tuberosity; **pp**, pubic peduncle. Scale bar equals 10 cm.

**Figure 8 pone-0024487-g008:**
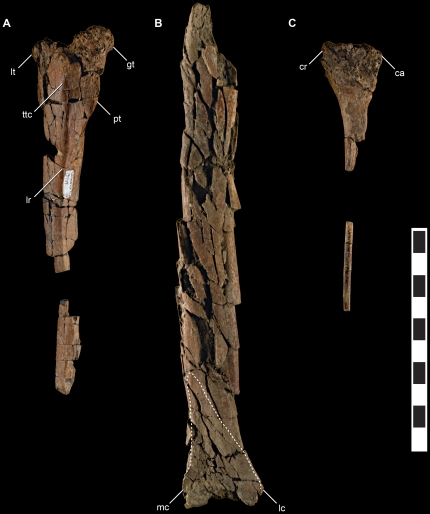
Femur, tibia, and fibula of *Talos sampsoni* (UMNH VP 19479). Left femur in lateral view (**A**); left tibia in cranial view (**B**); left fibula in lateral view (**C**). Dotted line illustrates impression of ascending process of ipsilateral astragalus. Abbreviations: **ca**, caudal aspect; **cr**, cranial aspect; **gt**, greater trochanter; **lc**, lateral condyle; **lr**, lateral ridge; **lt**, lesser trochanter; **mc**, medial condyle; **pt**, posterior trochanter, **ttc**, lateral tubercle of trochanteric crest. Scale bar equals 10 cm.

**Figure 9 pone-0024487-g009:**
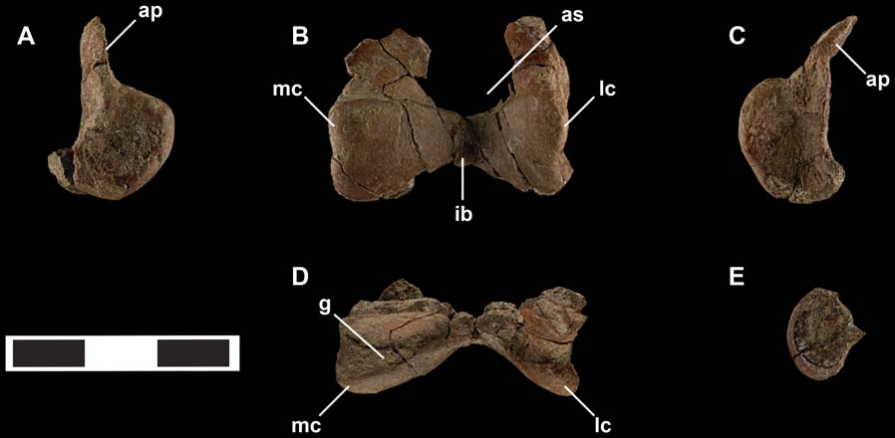
Tarsal elements of *Talos sampsoni* (UMNH VP 19479). Left astragalus in medial (**A**), cranial (**B**), lateral (**C**), and proximal (**D**) views; left calcaneum in lateral view (**E**). Abbreviations: **ap**, ascending process; **as**, astragalar sulcus; **g**, groove at base of ascending process; **ib**, intercondylar bridge; **mc**, medial condyle; **lc**, lateral condyle. Scale bar equals 3 cm.

**Figure 10 pone-0024487-g010:**
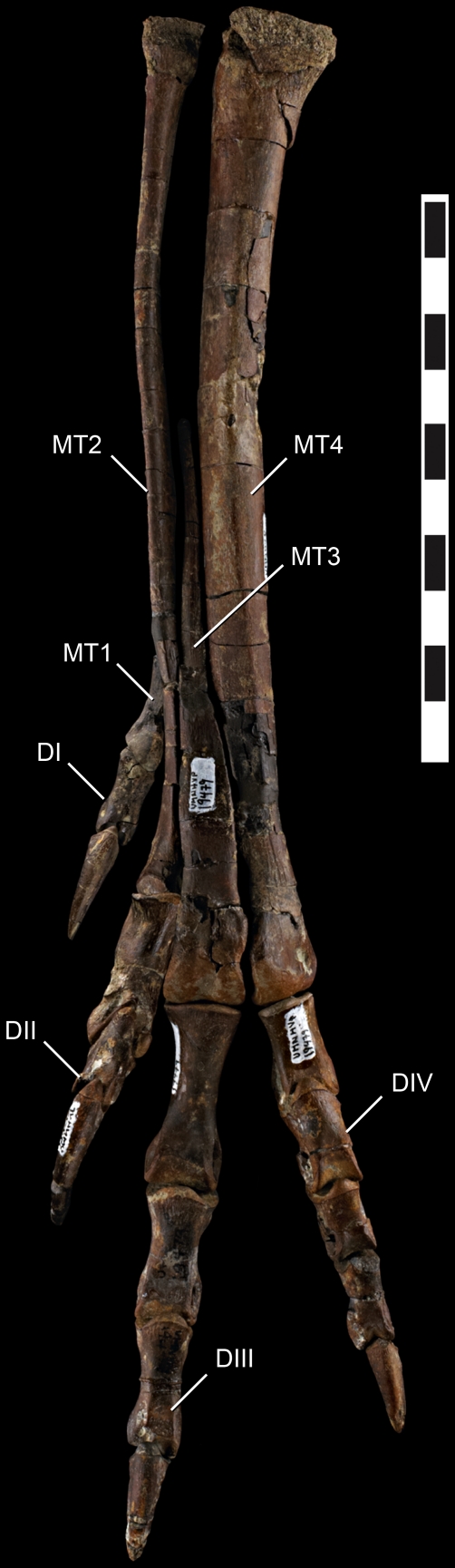
Articulated pes of *Talos sampsoni* (UMNH VP 19479). Digit one reversed from right pes. Abbreviations: **DI**, digit one; **DII**, digit two; **DIII**, digit three; **DIV**, digit four; **MT1**, metatarsal one; **MT2**, metatarsal two; **MT3** metatarsal three; **MT4**, metatarsal four. Scale bar equals 10 mm.

**Figure 11 pone-0024487-g011:**
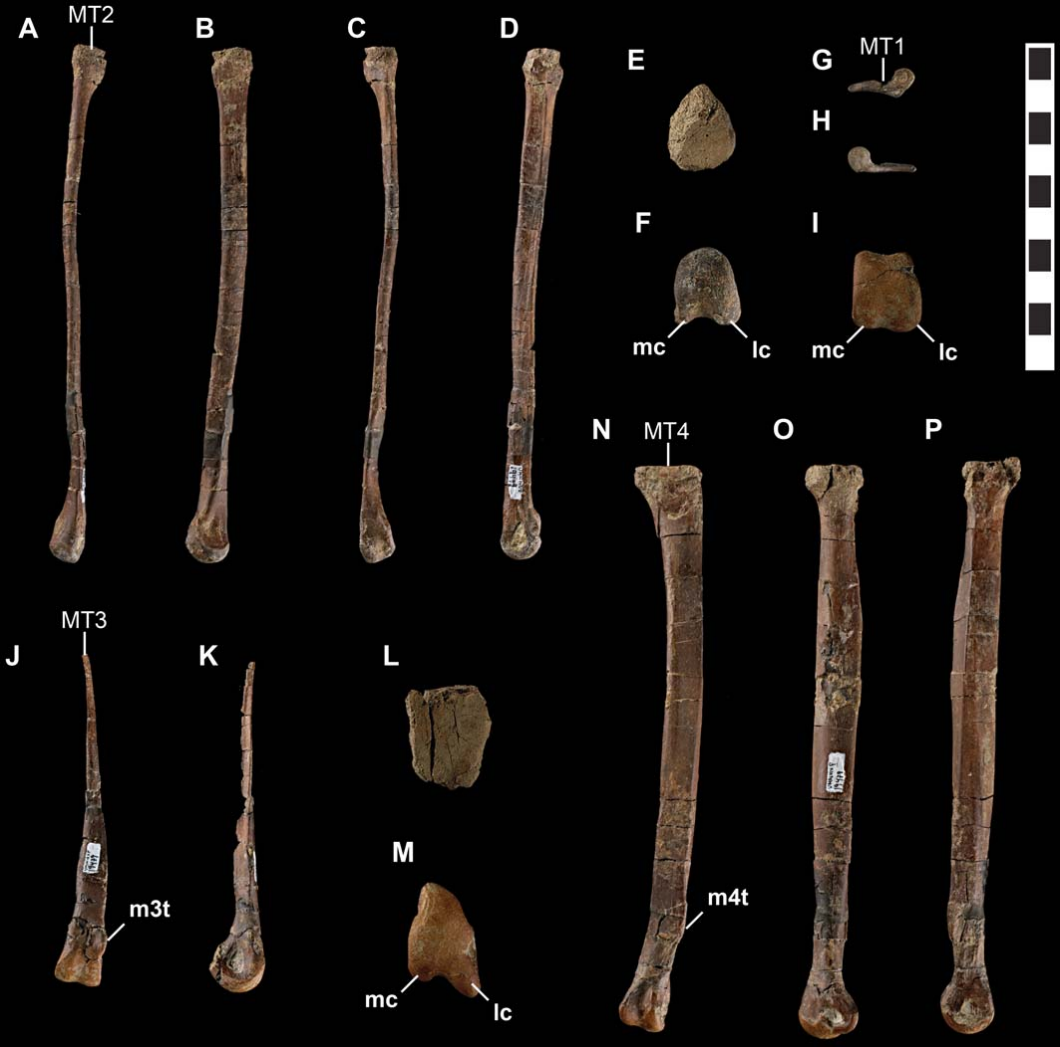
Metatarsus of *Talos sampsoni* (UMNH VP 19479). Right metatarsal I in lateral (**G**) and medial (**H**) views; left metatarsal II in extensor (**A**), medial (**B**), flexor (**C**), lateral (**D**), proximal (**E**), and distal (**F**) views; left metatarsal III in extensor (**J**), medial (**K**), and distal (**I**) views; left metatarsal IV in flexor (**N**), lateral (**O**), medial (**P**), proximal (**L**), and distal (**M**) views. Abbreviations: **lc**, lateral condyle, **mc**, medial condyle; **m3t**, tab on extensor surface of metatarsal three; **m4t**, tab on flexor surface of metatarsal four; **MT1**, metatarsal one; **MT2**, metatarsal two; **MT3** metatarsal three; **MT4**, metatarsal four. Scale bar equals 10 mm.

**Figure 12:**
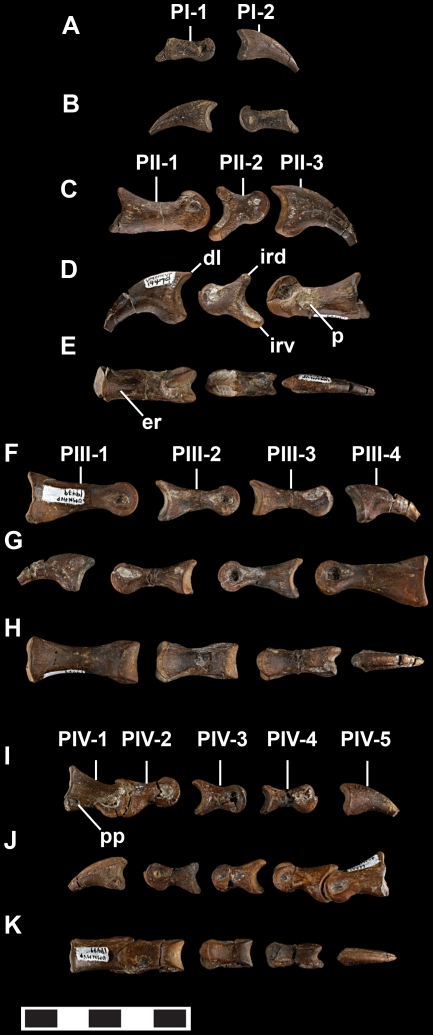
Pedal phalanges of *Talos sampsoni* (UMNH VP 19479). Digit one of right pes in lateral (**A**) and medial (**B**) views; digit two of left pes in medial (**C**), lateral (**D**), and extensor (**E**) views; digit three of left pes in medial (**F**), lateral (**G**), and extensor (**H**) views; digit four of left pes in medial (**I**), lateral (**J**), and extensor (**K**) views. Abbreviations: **er**, extensor ridge; **dl**, dorsal “lip”; **ird**, dorsal intercondylar ridge; **irv**, ventral intercondylar ridge; **p**, pathology; **pp**, proximal pit; **PI-1**, first phalanx of digit 1; **PI-2**, second phalanx of digit 1; **PII-1**, first phalanx of digit 2; **PII-2**, second phalanx of digit 2; **PII-3**, third phalanx of digit 2; **PIII-1**, first phalanx of digit 3; **PIII-2**, second phalanx of digit 3; **PIII-3**, third phalanx of digit 3; **PIII-4**, fourth phalanx of digit 3; **PIV-1**, first phalanx of digit 4; **PIV-2**, second phalanx of digit 4; **PIV-3**, third phalanx of digit 4; **PIV-4**, fourth phalanx of digit 4; **PIV-5**, fifth phalanx of digit 4. Scale bar equals 5 mm.

#### Remarks

We estimate *Talos* at approximately two meters in total length, with a body mass of approximately 38 kg based on distal tibia width [49]. Select measurements of UMNH VP 19479 are provided in Table S1.

At face value, the pes of *Talos* appears notably more gracile than *Troodon*. To quantify this observation we investigated proportional differences between metatarsal elements of *Talos* and a growth series of *Troodon* by calculating linear regressions and p-values (F test) for length-to-width ratios of metatarsals II and IV (Figure 4A–D). Although sample sizes were limited (3–5 representative elements per regression), *Troodon* specimens representing smaller and larger elements were available in all cases. The length-to-transverse width (p<0.05) and length-to-craniocaudal width (0.05<p<0.1) ratios of metatarsal II and length-to-craniocaudal width of metatarsal IV (p<0.05) of *Talos* are markedly lower than estimated for comparable metatarsals of *Troodon*. However, the length-to-transverse width ratio of metatarsal IV falls within the variation observed for *Troodon*. This finding supports the interpretation that discrepancy in the slenderness of metatarsal II of *Talos* as compared to *Troodon* is a distinguishable feature and is not simply attributable to ontogenetic variation. These data also confirm that the metatarsus of *Talos* is significantly more gracile than that of *Troodon*, even among smaller and more immature individuals.

### Description and Comparisons

#### Axial Column

The axial column is represented by a single, nearly complete dorsal vertebra, two fragmentary dorsal centra (deriving from the middle and caudal aspects of the series), an unfused sacral centrum, a caudal vertebral fragment, multiple proximal chevron fragments, and additional poorly preserved surface fragments of dorsal and sacral(?) vertebrae.

The oval centrum of the nearly complete dorsal vertebra together with a lack of a hypapophysis suggests a middle position in the dorsal series. This centrum is amphicoelous and taller than wide, bearing a degree of ventral constriction consistent with its interpretation as a mid-dorsal. Postmortem distortion and root rot has obscured much of the cranial and right lateral aspects of this vertebra, including the shape of the transverse processes, both prezygapophyses, and associated laminae. An isolated dorsal centrum, identified as belonging to the caudal part of the series, is platycoelous and spool-shaped, lacking any ventral constriction.

As in *Troodon* (e.g., MOR 553S-8.20.92.305) and *Saurornithoides* [2], yet unlike other troodontids [2], the neural arch of the mid-dorsal appears pneumatic (Figure 5B). However, the degree and position of pneumatic features is difficult to quantify due to incomplete preservation. Whereas pneumatic foramina appear to be present within infrapostzygapophyseal fossae, it is unclear if the infraprezygapophyseal fossae are also perforated. The ventral surfaces of the transverse processes and all preserved dorsal centra are apneumatic as in *Troodon*, *Saurornithoides*, *Byronosaurus*, *Sinovenator*, and *Mei* [32], [50].

The single preserved mid-dorsal neural spine is long as in *Troodon* (MOR 553S-8.20.92.305) (Figure 5A–B) and unlike the relatively short, fan-shaped spines of basal troodontids [5], [13]. The neural spine tapers to a dorsocaudal point in lateral profile (Figure 5A), a morphology not exhibited by other troodontids. It is possible that this is a preservational artifact related to weathering of the distalmost spine, although it may also prove diagnostic with the collection of additional materials. The middle dorsal neural spines of *Troodon* (MOR 553S-8.20.92.305, 8.28.92.270) are subrectangular, yet do exhibit a slight caudodorsal slope. The dorsal margin of the neural spine of *Talos* is transversely expanded on the left side and flush on the right. This discrepancy is likely attributable to weathering and crushing of the spine and it is therefore unclear if *Talos* possesses the spine-table of *Troodon*.

In contrast to *Saurornithoides*, the dorsal margin of the parapophyses are projected from the neural arch. A similar condition is evident on *Mei*. Projection of the dorsal periphery of the parapophyses may reflect incipient development of the stalk-like condition in dromaeosaurids [13], [51] (Figure 5B). The neural canal is enlarged as in other paravians [34] (Figure 5B). Postzygapophyseal facets are flat, subcircular, and angled approximately 45° from the sagittal axis.

The unfused sacral centrum is platycoelous, possessing one slightly concave kidney-shaped articular facet and one subcircular flat facet and either represents the first (S1) or penultimate (S5) sacral vertebra (Figure 5C). The ventral margin is broadly convex, lacking a ventral groove. The neural arch was not yet fused to the centrum and is not preserved.

A largely damaged fragment is the sole representative of the caudal series. The fragment indicates that the distal caudal centrum was elongate, subrectangular, and transversely narrower ventrally than dorsally. There is some indication that the ventral aspect of the centrum may have possessed a groove. The neural arch is excised by a longitudinal sulcus in lieu of a neural spine—a synapomorphy of Troodontidae [26]. Chevron fragments display a cylindrical ventral shaft (as in *Saurornithoides* and *Troodon* (NMC 12340))[26].

#### Forelimb

The ulna is relatively straight shafted, bowing slightly at its proximal extent. The proximal aspect is subtriangular and, although damaged, appears to have possessed a weak olecranon process as in other troodontids (e.g., *Sinornithoides*, *Troodon*; MOR 553S-8.12.92.219) (Figure 6). Only the distalmost aspect of the proximal articular facet is preserved so it is unclear if a ridge would have delineated the facet as in other paravians. The shaft is generally smooth; however, just distal to the proximal articular facet a flange marks a well-defined origination point for a slight muscle scar that trends along the extensor surface of the shaft for nearly its entire length, as in *Troodon formosus* (NMC 12340) [26]. A lateral interosseous ligament scar is not visible but can be felt on the shaft. The distal end is transversely expanded, angled at approximately 45 degrees from the transverse plane of the proximal articular facet, and exhibits a weakly convex articular surface (Figure 6B). One ∼25 mm fragment of limb bone shaft may represent the only known portion of the radius.

#### Pelvis

The ilia are severely fragmented and crushed, and only a portion of one acetabulum is identifiable. No additional fragments of ilia preserve natural margins. The right and left pubes are represented solely by portions of the shafts (Figure 7F–G), the longest preserving approximately 122 mm of the total length. Proximally the shafts are transversely compressed; distally they transition to a sub-triangular cross-section. Proximal to the pubic apron, each pubic shaft bows laterally to meet the ipsilateral ilium. However, in lateral profile, the pubic shafts appear relatively straight as in other troodontids [5], [32] (Figure 7F–G).

Due to proximal damage it is unclear if *Talos* exhibited a near-propubic to propubic pelvis as in most troodontids (e.g., *Troodon* (MOR 246) *Saurornithoides*, *Sinusonasus* [52], and *Sinornithoides*), or a retroverted pubis as in the basal taxon *Sinovenator* [5]. The distal aspect of the pubes are also not preserved, thus it cannot be determined if *Talos* possessed a pubic boot as in *Troodon* (MOR 553S) or lacks this feature as in *Sinovenator* [5] and *Sinornithoides* [53]. Although the absence of this feature in some troodontid specimens has also been attributed to skeletal immaturity [12].

The pubic apron is not preserved; however, broken surfaces reveal that the apron originated from the craniomedial aspect of the shaft as in *Sinovenator* (IVPP V12615) and *Troodon* (MOR 553S-8.3.9.387) (Figure 7F–G). It can also be determined that the pubic apron would have been restricted to less than half the length of the pubes as in *Troodon* (MOR 553S-8.3.9.387) and unlike the extensive pubic shaft exhibited by *Sinovenator*.

The ischia are represented by nearly complete right and partial left counterparts (Figure 7A–E). The acetabular margin of the proximal ischium is strongly concave rather than relatively flat as observed in *Saurornithoides* [32] (Figure 7B–C). Whereas its medial surface is flat, the lateral surface of the ischium is concave ventral to the caudodorsal margin. This configuration is observed in *Saurornithoides* [32], *Sinusonasus* [52], and *Troodon* (MOR 553L-7.27.8.87), yet not in more basal troodontids [53]. The presence of a caudodorsal ridge on the ischial shaft is shared with *Saurornithoides* and *Troodon* (MOR 553L-7.27.8.87) (Figure 7C) and may be homologous with the ridge dividing the lateral ischial shaft into cranial and caudal aspects in other paravians (IVPP V12615) [54]. The caudodorsal ridge appears to originate in a slightly more proximal position on *Saurornithoides* and *Talos* than it does on *Troodon* (MOR 553L-7.27.8.87); however because of small sample size this difference may also be attributable to ontogenetic and/or individual variation.

In contrast to *Sinovenator*, *Mei*, some dromaeosaurids, and the slight protuberance on *Troodon* (MOR 553L-7.27.8.87), there is no well-defined proximodorsal tuberosity (sensu [55]) on *Talos*. However, this region is faintly rugose (Figure 7B) and could therefore represent incipient development of the proximodorsal tuberosity related to either ontogenetic stage or phylogenetic position. Although damaged, the overall morphology of the ischial shaft compares closely with *Troodon* (MOR 553L-7.27.8.87) and *Saurornithoides* [32], suggesting that the obturator process would have been located near midshaft. A small isolated section of the ischium appears to represent the shaft terminus. However, damage to the periphery of this fragment makes its exact morphology and position indeterminate.

#### Hind limb. *Femur .*

Only a portion of the proximal aspect of the left femur is preserved and the proximal-most aspect of this fragment is eroded (Figure 8A). A robust, crest-like posterior trochanter extends from the caudolateral aspect of the proximal shaft (Figure 8A). The trochanter is notably more robust than on specimens of *Troodon*, even among larger individuals (MOR 553S-7.28.91.239, 7.16.0.61, MOR 748). *Talos* appears to have lacked a pronounced fourth trochanter as in *Saurornithoides* (AMNH FR 6516), *Troodon* (e.g., MOR 553S-7.16.0.61; 7.28.91.239), and *Sinornithoides* [12]. However, due to incomplete preservation of the proximal femur, this cannot be determined with certainty.

A small pointed tubercle (homologous with the trochanteric crest) [56] extends from the lateral aspect of the proximal femur in *Talos* (Figure 8A), *Troodon* (MOR 553S-7.28.91.239), *Saurornithoides* [32], and *Sinornithoides* [12]. As in *Saurornithoides* [32] and *Sinornithoides* [12], a ridge originates just distal to this tubercle and extends distally along the lateral aspect of the shaft (lateral ridge, sensu [51]) (Figure 8A). A distinct lateral ridge is present and well developed on some, yet not all individuals of *Troodon*. The exact morphology of the greater and lesser trochanters and femoral head cannot be assessed due to damage. However, the cross-section approximates the sub-triangular morphology typical of troodontids, exhibiting a concave cranial margin between the femoral head and greater trochanter in proximal view.

#### *Tibia*

A partial left tibia is preserved (Figure 8B). The proximal aspect of the tibia was not recovered and the remainder of the element was shattered upon surface exposure. A depression on the cranial face of the distal tibia indicates that an extensive ascending process of the astragalus was present and covered the entire transverse surface of the tibia as in *Zanabazar* [32]. The ascending process of the astragalus was tall (approximately 63 mm in dorsoventral extent), trended medially (as in *Troodon*, MOR 553-7.7.91.19), and tapered proximally toward the craniomedial aspect of the tibial shaft (Figure 8B). The medial aspect of the distal tibia is craniocaudally expanded relative to the lateral side, as in theropods generally. There is little evidence of contact with the fibula as in *Sinornithoides* [53], yet unlike the condition in *Zanabazar* on which a large, striated fibular fossa is present [32]. The lateral face of the distal tibial shaft is flattened.

#### *Fibula*

Only a portion of the proximal fibula and a small section of the fibular midshaft are preserved (Figure 8C). The proximalmost aspect of the fibula is damaged and its morphology cannot be assessed. The internal surface is flat and lacks a pronounced fibular fossa as in *Troodon* (e.g., MOR 553S-8.17.92.265) and other paravians. The proximal shaft bows medially to contact the tibia. The shaft is oval in cross-section and narrow in diameter.

#### *Tarsus*

Tarsal elements of *Talos* are represented by a nearly complete left calcaneum and a nearly complete left astragalus missing the proximal ascending process, the medial condyle of the right astragalus, and a fragment of the right calcaneum. The astragalus and calcaneum are entirely unfused on both sides.

Medial and lateral astragalar condyles are well developed and connected by a highly constricted intercondylar bridge, forming an unusually deep v-shaped outline in cranial (Figure 9B) and proximal (Figure 9D) views. Constriction of the intercondylar bridge is more extreme than that observed in *Troodon* (e.g., CMN 1560, CMN 199, MOR 553S-11.1.01.1), *Zanabazar junior* (IGM 100/1), *Sinornithoides youngi* (IVPP V9612), *Sinovenator* (IVPP V12615, V12583), or *Mei*. The astragalar condyles are subequal in their cranial extent (Figures 3B and 9D) unlike *Sinovenator*, *Sinornithoides,* and *Troodon* (e.g., MOR 553S-8.20.92.311, 7.7.91.19), in which the medial condyle extends further cranially than its lateral counterpart.

As in *Troodon* (e.g., MOR 553S-8.20.92.311, 7.7.91.19) and other troodontids [32], [57] the medial condyle is transversely wider and less proximally extensive in cranial view (Figures 3B and 9B). This low profile on *Talos* corresponds to a relatively horizontal proximal margin, similar to *Sinovenator* (IVPP V12583). In contrast, the proximal margin of the medial condyle of *Sinornithoides* and *Troodon* (CMN 1560, CMN 199, MOR 553S-11.1.01.02) is strongly convex and bulbous.

As in *Sinornithoides* and *Sinovenator* (IVPP V12583), *Talos* lacks a groove across the craniodorsal face of the astragalus. This differs from *Troodon* (CMN 1560, CMN 199; MOR 553S-8.20.92.311, 7.7.91.19, 11.1.01.02, 11.1.01.1) and *Mei*, which exhibit a cranial or craniodorsal groove. In medial view, *Troodon* possesses a medial condyle with a rounded profile that approaches the base of the ascending process or extends proximal to it. In contrast *Talos* has an asymmetrical medial condyle that slopes distally from the base of the ascending process (Figure 9A).

The groove demarcating the contact between the astragalar body and ascending process forms an unusually wide trough in *Talos* (Figure 9D) and *Troodon* (e.g., MOR 553S-8.20.92.311, 7.7.91.19). However, the trough is straight on *Troodon*, whereas it is v-shaped on *Talos* due to the hyperconstricted intercondylar bridge. This groove is not well defined on the juvenile troodontid specimen IVPP V10597 [57]. In addition, *Troodon* specimens have a well-developed notch between the lateral condyle and the ascending process that is nearly absent on *Talos* (Figure 9C).

A deeply depressed sulcus excises the cranial surface of the ascending process on *Zanabazar* [32] and is variably expressed on *Troodon* (NMC 199; MOR 553S-8.20.92.311, 7.7.91.19), yet is absent on the juvenile troodontid specimen IVPP V20597 [57]. Although the mid-portion of the ascending process is damaged on *Talos*, preserved morphology suggests the presence of a similar depression (Figure 9B). However, due to damage, it cannot be determined if the ascending process of *Talos* exhibited the rounded tuberosity that characterizes the cranial surface of the ascending process in *Zanabazar* [32] and *Troodon* (e.g., NMC 199; MOR 553S-8.20.92.311, 7.7.91.19). The corresponding aspect of the ascending process of the astragalus just proximal to the medial condyle is markedly thickened on *Talos* (Figure 9A). In contrast, this area is craniocaudally restricted on *Troodon* (MOR 553S-8.20.92.311, 7.7.91.19).

The calcaneum is a transversely compressed, medially concave, disk-shaped element that tapers to a point at its caudal aspect (Figure 9E). The peripheral margin is transversely thickened at its cranial extent to meet a corresponding notch in the lateral condyle of the astragalus. As in IVPP V10597 [57], there appears to have been little contact with the fibula.

Currie and Peng [57] suggested that the calcaneum in troodontids is lost rather than fused as was reported by Russell [26]. The extremely small and slender nature of the unfused calcaneum of *Talos* and *Sinovenator* (IVPP V12583), the presence of a partially fused calcaneum on one large *Troodon* astragalus (MOR 553S-11.1.01.1), and an unfused theropod calcaneum (CMN 1791) referred to *Troodon* that compares favorably with the morphology exhibited by *Talos* confirm the presence of a calcaneum in several troodontid species.

Interestingly, the preserved fragment of the right astragalus is slightly larger than the corresponding aspect of the left astragalus. We therefore scanned the left tarsal elements with MicroCT to rule out potential pathological modifications resulting from the injury to the second pedal digit. Although it is conceivable that this difference is associated with trauma to the left pes, no evidence of internal post-traumatic reorganization or cortical proliferation was observed in any tarsal element.

#### *Metatarsus*

The metatarsus of *Talos* is asymmetrical—a synapomorphy of Troodontidae [26], [32], [58]—being comprised of a robust fourth metatarsal, proximally pinched third metatarsal (arctometatarsalian), and markedly compressed, proximodistally abbreviated second metatarsal (Figure 10). The left metatarsus is well preserved, missing only metatarsal I, the proximalmost shaft of metatarsal III, and exhibiting slight damage to the proximal articular surfaces of metatarsals II and IV. The right metatarsus is less complete, preserving metatarsal I, yet missing the proximal and distal ends of metatarsal III and most of the distal aspect of metatarsal II.

Relative to *Troodon* and other derived troodontids, the metatarsus of *Talos* is significantly more slender in proportion, including *Troodon* specimens of smaller size and younger ontogenetic stages (e.g., MOR 553E-7.1.9.9) [9]. The extreme slenderness of the metatarsus of *Talos* relative to *Troodon* is attributable to reduction of the second metatarsal (in both craniocaudal and transverse dimensions; Figures 4A–B) and the fourth metatarsal in craniocaudal dimension only (Figure 4C). The transverse width of the fourth metatarsal (Figure 4D) is in proportion with the ontogenetic series of *Troodon*.

Metatarsal I is diminutive, with a laterally compressed, attenuated shaft and an asymmetrical, subtriangular, and bulbous distal articular condyle that projects medially, approximately 30° from the main axis of the metatarsus (Figure 11G–H).

Metatarsal II is markedly reduced in length and breadth, consistent with a reduced capacity for weight bearing as is typical for deinonychosaurians [59]. Its shaft is transversely compressed to such a degree as to be nearly splint-like in relative proportions, exhibiting a midshaft length-to-width ratio approximately one half that of *Troodon* (e.g., MOR 553S-7.8.91.28, 7.18.92.5). Metatarsal II terminates in a transversely compressed, gently rounded articular surface comprised of a lateral condyle approximately twice the size of the medial condyle (Figures 11A and 11F). In contrast, metatarsal III exhibits proportionally large, subtly ginglymoid articular condyles with a poorly developed extensor fossa (Figures 11I and 11J).

As in other deinonychosaurians (e.g., *Velociraptor*) [51], yet in contrast to *Saurornithoides* [32], the distal articular surface of metatarsal III is asymmetrical, exhibiting a larger medial condyle (Figure 11I). Proximal to the distal condyles, the shaft of metatarsal III broadens transversely before attenuating to less than 5 mm in size, with a subtriangular cross section throughout (Figure 11J). The dorsolateral aspect of the shaft of metatarsal III just proximal to the distal condyles is sinuous and marked by a pronounced bony “tab” that extends laterally to fill an indentation on the dorsomedial margin of metatarsal IV (Figures 3C and 10). This feature is poorly developed (MOR 748; MOR 553S-7.29.92.113, 8.6.9.406; NMC 12340) on specimens referred to *Troodon* (except TMP 92.36.575), which instead display a gently concave dorsolateral margin. A bony tab on metatarsal III is apparently also absent on *Saurornithoides mongoliensis* (AMNH FR 6516) and *Sinovenator* (IVPP V12615). This feature may be present on the primitive troodontid IGM 100/44, although it does not appear to characterize the better-preserved metatarsal elements of this taxon (fig.2 in [60]).

In similar fashion, a bony flange extends from the ventromedial aspect of the shaft of metatarsal IV just proximal to the distal condyles and underlies the shaft of metatarsal III, bracing it ventrally (Figure 11N). On *Troodon*, this feature is robust and proximally continuous, extending up the shaft to nearly exclude metatarsal III in ventral view and causing the shaft of metatarsal IV to appear strongly kinked laterally at its distalmost extent (MOR 748; TMP 92.36.575). In more primitive troodontids (e.g., *Sinornithoides*, *Sinovenator*, *Mei*) this flange is absent. In *Talos*, the feature is notably less pronounced, expressed as a convex tab of bone, and does not extend up the ventral shaft to abut metatarsal II. Although the feature is considered taxonomically significant for *Talos*, its degree of development may also be ontogenetically variable as a single smaller *Troodon* specimen (MOR 553S-8.17.92.260) exhibits a similarly reduced condition.

In dorsal view, a small protuberance marks the dorsodistal corner of the lateral collateral ligament pit on the fourth metatarsal, creating a notch along the lateral aspect of the lateral distal condyle (Figure 3D). This feature is shared between *Talos* and *Troodon*. However, in the latter taxon the notch is more pronounced and extends to the distal margin in dorsal view (MOR 553L-7.28.8.102; MOR 553S-11.1.01.8), whereas in *Talos*, a bony extension closes off this notch, creating a rounded pit (Figure 3D). Metatarsal IV of both taxa exhibits a strong lateral bow (Figure 11N).

#### *Pedal Phalanges*

All phalanges of the left foot are preserved except the proximal phalanx of the first pedal digit (although IV-1 and IV-2 are presently inseparable), whereas phalanges II-1, II-3, III-4, and IV-3 are missing from the right foot.

Phalanx I-1 is relatively straight shafted with a concave proximal articular facet bearing an asymmetrically oriented, laterally extensive heel, asymmetrical distal condyles, and a deep lateral collateral ligament pit (Figure 12A). Phalanx I-2 trends medially and possesses asymmetrical lateral and medial grooves, the lateral skewed further dorsally (Figure 12A–B).

**Figure 13:**
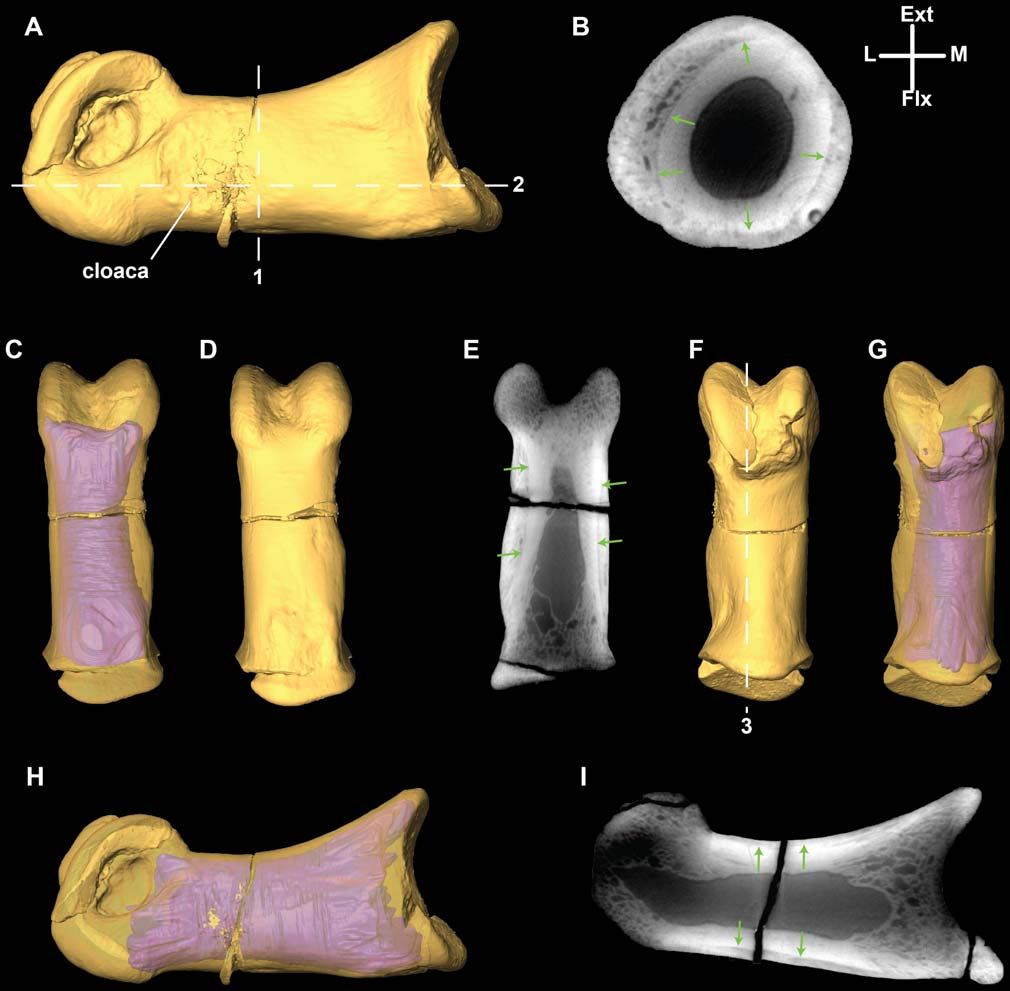
Micro-CT renderings of the pathological first phalanx of the left second pedal digit (II-1) of *Talos sampsoni* (UMNH VP 19479). Phalanx II-1 in lateral (**A**, **H**), flexor (**C**–**D**). extensor (**F**–**G**) views. Cross-sectional images of *Talos* taken through axial (**B**), transverse (**E**), and parasagittal (**I**) planes. Hashed lines in A and F represent the plane of section for images in B (1), E (2), and I (3). Anatomical reference lines show position of axial cross section (B) only. Green arrows in B, E, and I depict the boundary between the original cortical contour (shown in purple on C, G, and H) and the exostotic bone. Abbreviations: **bc**, bony collar; **Ic**, lateral condyle; **mc**, medial condyle; **oc**, original cortical surface.

As is characteristic of deinonychosaurians, phalanges of the second digit are modified to support an enlarged, trenchant ungual (Figure 12C–E). Phalanx II-1 of *Talos* is the most distinctive, bearing a robust and straightened shaft (Figure 12C–D). The proximal articular facet is oval in shape, lacking a medial ridge and exhibiting a rudimentary ventral heel. Just distal to the proximal articular facet the dorsal aspect of the shaft is pinched into a raised ridge (Figures 12E). In marked contrast to the highly symmetrical and circular distal condyles of other troodontids, the craniodorsal aspect of the lateral condyle of phalanx II-1 is flat, creating a distally pointed profile in lateral view (Figure 12D). The medial condyle exhibits a similar, yet less angular, outline. The lateral collateral ligament pit on this phalanx is asymmetrically enlarged, with the ventral and proximal margins of the pit extended in their respective directions (Figure 13A).

Notably, phalanx II-1 of *Talos* exhibits extreme pathological modification and several aspects of its morphology cannot be considered representative for the species. A surface lesion, demarcated by a narrow raised edge of cortical bone, is visible just proximal to the lateral collateral ligament pit (Figure 13A). MicroCT analysis of phalanx II-1 reveals an extensive accumulation of cortical bone consistent with post-traumatic or post-infection exostosis (Figures 13B–C and 13G). The internal bony organization of II-1 has also been modified in accordance with these external alterations. Reorganization is particularly pronounced in the distal half of the element in specific association with the subcortical condylar areas (see additional discussion of osteopathology below). Thus, the unusually straight shaft and highly asymmetrical distal condyles are clearly attributable to secondary osteogenesis.

Phalanx II-2 is generally similar to other deinonychosaurians. It is compact, and in contrast to phalanx II-1, the proximal articular facet exhibits a pronounced median ridge and markedly well-developed heel (Figure 12C–E). The medial distal condyle shares the asymmetrical outline of phalanx II-1 to a minor degree (the lateral condyle is incomplete along the dorsal margin). In contrast to the situation in phalanx II-1, there are no external signs of osteopathology on II-2. Moreover, a micro-CT based comparison of the left and right II-2 reveals near identical internal organization. Phalanx II-3 has asymmetrical grooves, a hint of a caudodorsally projecting lip (as evidenced by a slight depression between the main body of the ungual and the dorsal aspect of the proximal articular facet when viewed laterally), and a moderately developed flexor tubercle (Figure 12C–D).

Phalanges of the third pedal digit of *Talos* exhibit proximoventral heels, weak (III-3) to absent (III-1, III-2) intercondylar ridges, and generally lack extensor pits (Figure 12F–H) (an extensor pit is slightly expressed on PIII-1). Lateral collateral ligament pits are subcircular and deep, whereas medial pits are shallower and proximodistally elongate (Figure 12F–G). The medial distal condyle of phalanx III-3 is slightly asymmetrical in circumference, mirroring the condition of II-I and II-2. The distal condyles are also asymmetric in overall size, the lateral being more robustly developed. This asymmetry is consistent with the modified weight-bearing role of this digit and is not attributable to pathology.

All phalanges of the fourth pedal digit lack extensor pits and possess shallower lateral collateral pits (Figure 12I–K). The proximal three phalanges have well-developed heels, the medial aspect of which is more robust. Phalanx IV-1 exhibits a concave proximal facet (taller than wide), with a medially inclined dorsal aspect. The medial distal condyle of phalanx IV-4 is more robust than the lateral. The proximal articular surfaces of phalanges IV-3 and IV-4 are subtriangular and relatively symmetrical. Two pronounced pits incise the ventrolateral and ventromedial aspects of phalanx IV-1 close to the proximal articular facet and dorsal to the planar heels. Only a ventrolateral pit is present on phalanx IV-2 and both pits are absent on IV-3 and IV-4. A similar trait is seen on the phalanges of the third digit, which also exhibit well-developed heels; however this feature is expressed as a slight depression on these phalanges in contrast to a distinct pit. These depression are distinct on both sides of phalanx III-1, the lateral aspect of III-2, and both lateral and medial aspects of III-3.

#### Histological description. *Proximal femur .*

The femoral sample (Figure S1) consists of only a small portion of the overall cross-section. The sample is 2.8 mm wide by 3.7 mm radially. A large portion of the inner cortex (approximately 60% of the entire sample) appears diagenetically altered, obscuring much of the original bone histology. A small area consisting of circumferentially organized lamellar bone with possibly one or two widely spaced lacunae represents the only visible portion of endosteal bone. Small patches of adjacent tissue exhibit a more varied extinction pattern and more numerous and randomly oriented lacunae. Examination of the remainder of the inner cortex under cross-polarized light reveals a faint pattern suggestive of fibro-lamellar bone.

The outer 40% of the cortex is better preserved, yet also displays evidence of diagenetic alteration. Mineral precipitants are concentrated at and around the central canal of primary osteons. Nevertheless, this region clearly consists primarily of fibro-lamellar bone with longitudinally arranged osteons. The extinction pattern under cross-polarized light differs from that expected in unaltered bone, and instead large, more irregular patches of extinction suggest crystal growth and replacement.

Three circumferential bands of cellular, lamellar bone divide the fibro-lamellar region. The middle and outer bands exhibit parallel extinction patterns consistent with circumferentially arranged crystals and lamellar bone. Greater recrystallization towards the cortex interior likely eliminates or obscures this extinction pattern from being fully expressed by the innermost band. Each of these bands is approximately 60–75 µm and contains three or more closely spaced lines. Two circumferential rows of primary osteons lie inward from the innermost band and six osteonal rows separate this band from the next. Three additional rows occur between this middle band and the outermost one. These separations measure 600 and 220 µm respectively.

The periosteal surface has a scalloped outline reflecting the development of a single row of primary osteons exterior to the third lamellar band. These retain large and somewhat oval vessel cross-sections indicating that these osteons were not fully developed at the time of death.

#### *Proximal fibula*

**Figure 14:**
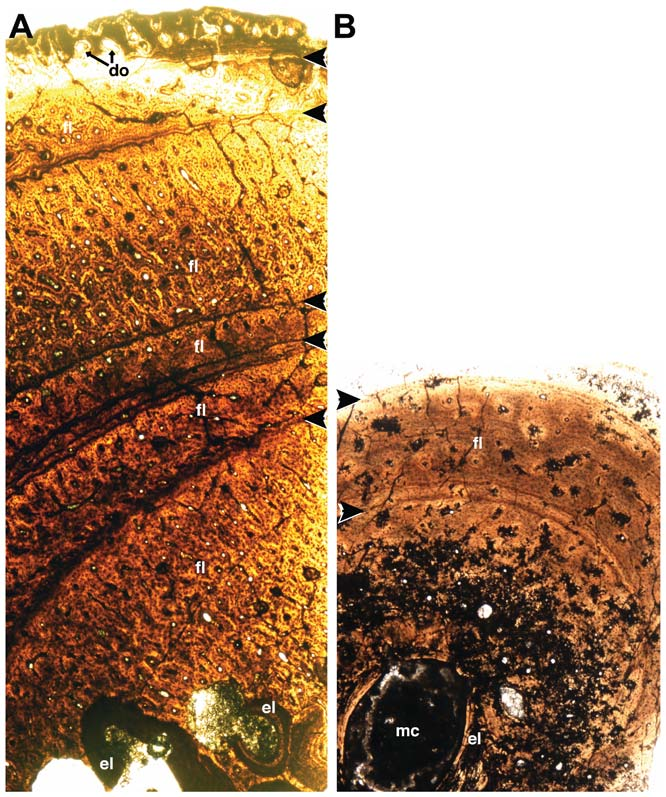
Proximal (A) and midshaft (B) histologic cross-sections of the fibula of *Talos sampsoni* (UMNH VP 19479). Medullary spaces are towards the bottom. Bands representing slowed growth including lamellar bone and lines of arrested growth are marked with “**<**”. The proximal section preserves five or six bands, whereas the midshaft has three. The third band of the midshaft section is not represented in this view due to taphonomic loss of the bone periphery. Between these bands, bone consists primarily of fibrolamellar bone with longitudinally oriented osteons. Episodes of endosteal deposition and erosion occur in the medullary regions of both sections. Abbreviations: **do**, developing osteons; **el**, endosteal deposition of lamellar bone; **fl**, fibrolamellar bone; **mc**, medullary cavity. Scale bars equal 0.3 mm.

The histologic sample of the proximal fibula (Figure 14A) represents an incomplete cross section 6.0 mm×4.1 mm in size, taken from the cranial aspect of the shaft. The overall geometry of the preserved tissues suggests that in comparison to the femur and fibula midshaft samples, the proximal fibula underwent greater shape changes during ontogeny.

The endosteal region of the proximal fibula preserves portions of at least six major growth intervals delineated by avascular lamellar bone. Also present are two large circular spaces lacking any distinct endosteal linings. These features exhibit a variety of crosscutting relationships relative to one another.

The majority of the 3.5 mm-thick cortex consists of fibro-lamellar bone with osteons exhibiting both longitudinal and radial organization. Five circumferential bands of various forms divide the cortex. Moving from the endosteal region outward, the innermost definitive band appears as a fuzzy gray zone approximately 50 µm thick. The second band (approximately 50–130 µm in thickness) consists of three lines that converge at either end of the sample. Osteons occur between the lines where separation exists. A single line, less than 25 µm thick, represents a third band and osteons are abundant beyond its periphery. The density of the spacing between osteons decreases towards the fourth band (approximately 50 µm thick), which consists of closely spaced lines, occasionally separated sufficiently to include an osteon. A fifth band (approximately 80 µm-thick) is marked by few osteons. Towards one end of the sample it continues as a series of closely spaced lines.

The four interspaces between these five definitive bands vary across the sample: 300–560 µm, 80–320 µm, 370–800 µm, and 240–500 µm, respectively. In general, the bands show parallel extinction consistent with the presence of lamellar bone. Overall, some portions of the cortex display higher osteonal density, suggesting more rapid growth in this direction across multiple bands. The periphery of the sample is marked by abundant osteons with wide vessel canals, suggesting the osteons were in an early stage of development.

#### *Fibula midshaft*

Due to post-fossilization bone loss, the cross-section of the fibular midshaft (Figure 14B) is incomplete and missing regions of the outermost cortex. The section is roughly oval in outline with long and short axes of 3.3 and 2.7 mm, respectively. The medullary cavity is similarly proportioned with its long axis parallel to that of the overall cross-section. As with the femur sample, considerable alteration, recrystallization, and replacement have occurred, particularly within the inner cortex. Additional smaller patches of altered bone are present in the middle and peripheral regions of the cross section. Adjacent to the medullary cavity, the cortex is comprised of two or three generations of crosscutting lamellar bone along the endosteal surface.

The cortex consists primarily of fibro-lamellar bone with longitudinally arranged osteons. This tissue differs from that of the femur, in that the osteons are positioned more randomly such that circumferential rows are not clearly developed. Those osteons close to the medullary cavity possess larger vessel spaces. As with the femur, the fibula midshaft cross-section does not exhibit an extinction pattern following the osteons, a typical feature of unaltered bone. Instead, the bulk of the cortex shows parallel extinction except where interrupted by two sets of lineations arranged at 110°–120° to each other. These lineations show parallel extinction and are significantly more prominent under cross-polarized light. Further, under high magnification (400x), the bone cortex exhibits a grainy, mosaic pattern suggesting significant recrystallization. This texture is absent from the endosteal bone, which exhibits a normal, parallel extinction pattern typical of lamellar bone.

Several, darkly stained circumferential lines or bands occur within the cortex. A pair of closely spaced lines lie about midway through the cortex. For most of the circumference these lines are approximately 70 µm apart; however, on both the lateral and medial aspects of the cortex, they converge to form a single line. Whereas elsewhere the coalesced or paired lines are outwardly convex, on one side they bend forming a concave stretch. Scattered osteons, circular or asymmetric, occur between the paired lines where separated. Both lines show small inward bends associated with more externally located osteons.

A second dark, 30 µm-thick band occurs more peripherally to the first set of paired lines. Measured at the point of greatest cortical thickness, the two are separated by approximately 510 µm. This band consists of two to three closely spaced lines, bends inwards to accommodate more external primary osteons, and is present throughout the cortex except where the sample is incomplete.

A third band also appears to be present, yet occurs only on a small portion of the cortex. Its absence elsewhere may be due to bone loss from the outer cortex. In the area where preserved, approximately 110 µm separate both the inner and middle bands and the middle and outer ones.

These three bands suggest zones of slowed growth. The bands lack parallel extinction typical of circumferentially arranged apatite crystals as in lamellar bone. However, the observed alterations to the bone may account for the absence of parallel extinction. The consistent deviation around potentially developing osteons as well as the parallel orientation of associated osteocyte lacunae suggests that these bands represent biologically significant signals.

### Pathology

Phalanx II-1 of the left foot exhibits extensive pathology consisting of both external and internal bony modifications (Figure 13). External pathologies include the presence of a surface opening (cloaca) at the distolateral margin of the shaft (Figure 13A), a circumferential bony collar developed along the length of the shaft (Figure 13B), and modification of the distal condyles. Although both condyles exhibit atypical (i.e., angular) peripheral margins, the dorsodistal margin of the lateral condyle appears distorted to the greatest extent. The bony collar is formed around the entire circumference of the phalanx, yet is preferentially developed along the lateral and flexor margins of the shaft, contributing to its distinctly straight appearance (Figures 12A and 12H– I).

MicroCT analysis reveals both the extent of the original cortical surface (Figure 13C and 13G) and the nature of internal remodeling within the element (Figures 13B, 13E, and 13H). It is apparent from the microCT results that the bony collar is comprised of disorganized bone around most of the peripheral portions of the outermost cortex and is morphologically consistent with bony callus formation (Figure 13B). Drainage and/or nutrient canals are visible passing from the medullary cavity through the cortical and exostotic bone to open on the external surface. Phalanx II-2 also exhibits subtle modifications of the articular surfaces (described above), but does not appear to possess any cortical or sub-cortical modifications.

Given that such trauma may have impacted function of the entire left hind limb, with the potential to induce morphological modifications in other elements in the series, we scanned additional phalanges, metatarsals, and tarsal elements of UMNH VP 19479. No evidence of abnormal cortical remodeling was identified outside the second pedal digit.

### Phylogenetic Analysis

**Figure 15:**
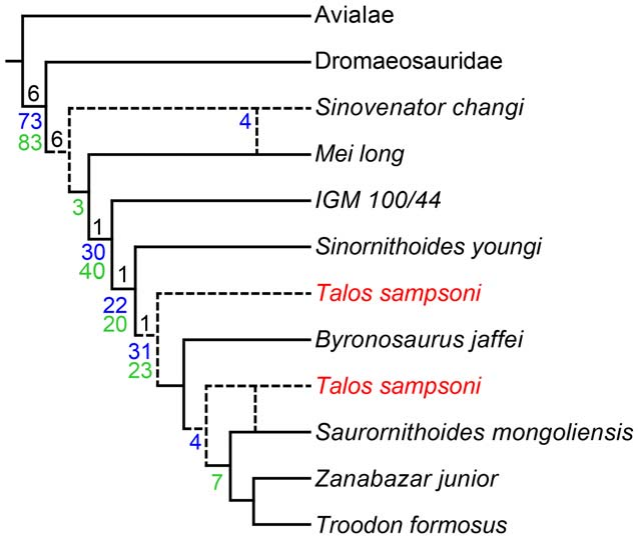
Evolutionary relationships of *Talos sampsoni* (in red). Tree illustrates combined topology of six MPTs (Tree Length = 48). Dashed lines indicate equally parsimonious positions for *T*. *sampsoni* (three alternates) and *Sinovenator changi* (two alternates). Node numbers represent support values (black = Bremer; blue = standard bootstrapping, GC values; green = symmetrical resampling, GC values).

Phylogenetic analysis recovers six MPTs varying in relationship between the basal troodontids *Mei long* and *Sinovenator changi*, and in the position of *Talos*. *Talos* is recovered in three equally parsimonious positions: as sister-taxon to the derived subclade containing *Byronosaurus*, *Saurornithoides*, *Zanabazar*, and *Troodon*; sister-taxon to *Saurornithoides*, *Zanabazar*, and *Troodon* only; and sister-taxon to *Saurornithoides mongoliensis* (Figure 15).

Nonetheless, in all topologies *Talos* is substantiated as a member of a derived, latest Cretaceous subclade of Troodontidae containing at least the taxa *Troodon*, *Saurornithoides*, and *Zanabazar*. This position is supported by the following traits: obturator process located at ischial midshaft, caudolateral ridge on ischial shaft, hypertrophied groove delineating the base of the ascending process of the astragalus, a kinked fourth metatarsal, and a notch in the dorsolateral aspect of the distal condyle the fourth metatarsal. However, none of these characters can be coded for *Byronosaurus*, therefore they may ultimately be found to characterize a more inclusive clade.

Only a single feature supports a monophyletic relationship among these taxa to the exclusion of *Byronosaurus*: the potential for *Talos* to possess a medially recurved dentary symphysis (which is absent in *Byronosaurus*). However, missing data are hardly evidence for a particular topological position. In addition, the hypothesized sister-taxon relationship between *Saurornithoides* and *Talos* recovered in two of the six MPTs is also supported by only a single feature—the presence of a caudodorsal ridge on the ischium that originates at the proximodorsal tuberosity (char. 40.1). This feature is unknown in several closely related forms (e.g., *Byronosaurus*, *Zanabazar*) and may prove to have a wider distribution within the clade. Moreover, the states of this trait may reflect individual or ontogenetic variation and may prove indistinguishable with the collection of additional specimens. Therefore there is little current phylogenetic support for any particular MPT in this study.

Not surprisingly, standard bootstrapping, symmetrical resampling, and Bremer values are low across the tree, further underscoring the general lack of support for the hypothesized relationships among derived troodontids (Figure 15). Lability of *Talos* primarily results from a lack of overlapping elements with other troodontids (specifically *Byronosaurus* and *Zanabazar*) and character conflict between *Talos* and *Troodon*. A comprehensive reevaluation of North American troodontid materials is required to resolve relationships among derived, large-bodied, Late Cretaceous troodontids and will likely add significant information toward the resolution of these issues, yet is clearly beyond the scope of this manuscript.

## Discussion

### Age assessment of *Talos sampsoni*

We used samples derived from the femur and fibula to conduct an age assessment of UMNH VP 19479 (Figure 14). All three samples display consistent histological features; each is comprised primarily of fibro-lamellar bone separated by at least three lines of arrested growth (LAGs). Interlag tissue exhibits several interwoven lines, as is common for non-avian dinosaurs [61], and few if any osteons. Where preserved, LAGs also display parallel extinction patterns consistent with lamellar bone.

Midshaft sections of the femur and fibula exhibit similar spacing between bands. A single row of incompletely developed osteons occurs peripheral to the last band in each of the three samples. The proximal fibula (Figure 14A) differs in possessing additional bands of diffuse coloration and a single line, as well as radially arranged primary osteons. Such differences may reflect greater ontogenetic changes in shape occurring in this area of the fibula.

All samples exhibit periods of faster growth represented by fibro-lamellar bone interrupted by slow growth periods as represented by the combination of lamellar bone and LAGs. Presuming the LAGs represent annually deposited features, UMNH VP 19479 was, minimally, in its fourth year of growth. If the circumferential bands within the proximal fibula reflect annual decelerations in growth and not simply remodeling reflecting shape change, the individual could be in its sixth year (Figure 14A).

The absence of secondary osteons and an external fundamental system [62], as well as the presence of the peripheral row of incompletely developed osteons, suggest that growth was ongoing in UMNH VP 19479. However, tighter spacing of increasingly peripheral bands of lamellar bone and LAGs indicate that overall growth was slowing. The number of growth bands with LAGs in UMNH VP 19479 contrasts with similarly sized, yet contemporaneous, *Troodon formosus* specimens from Montana [63]. For example, a *Troodon* tibia of similar length (320 mm; MOR 553S-7.20.91.132) exhibits only two growth lines. This suggests that *Talos sampsoni* would likely have plateaued at a significantly smaller size than *Troodon formosus*.

Observed fusion within this specimen is also consistent with a maturing animal, yet one that has not yet approached maximum size. Predominantly fused neurocentral sutures are present in some, yet not all dorsal vertebrae. Neurocentral sutures are unfused on at least one sacral vertebra and the sacrum has yet to form a cohesive unit. The calcanei and astragali also remain free. Sacral and tarsal fusion occurs in adult *Troodon formosus* individuals that express an external fundamental system [63].

### Kaiparowits troodontid materials

Previously, troodontid materials recovered from the Kaiparowits Formation were referred to *Troodon* based on the morphology of isolated teeth collected during microvertebrate surveys [64]–[65]. Over the past eight years, macrovertebrate surveys conducted by the Utah Museum of Natural History (UMNH), the Raymond Alf Museum of Paleontology (ALF), and the University of California at Berkeley Museum of Paleontology (UCMP) have produced additional troodontid remains from the Kaiparowits Formation including an isolated, left frontal (UMNH VP 16303), an isolated caudal vertebra (UMNH VP uncatalogued), a fragmentary partial skeleton (UCMP 149171), and several isolated teeth (UMMH VP 11806, UMNH VP 12507).

The troodontid frontal UMNH 16303 compares closely with those comprising the *Troodon formosus* hypodigm (e.g., AMNH 6174; TMP 79.8.1; MOR 553S-8.4.92.150, 7.15.0.41) in possessing an elongate, triangular morphology, an extensive orbital rim, a prominent ridge defining the rostral limit of the supratemporal fenestra, and a large, laterally extensive postorbital process [26]. However, differences can be observed among various specimens referred to *T*. *formosus* (AMNH 6174; TMP 79.8.1; MOR 553S-8.4.92.150, 7.15.0.41), as well as between UMNH VP 16303 and some of the specimens in the *T*. *formosus* sample. Variable features include the morphology of the orbital margin, supratemporal ridge, and orbital bulbs. These features may reflect ontogenetic and/or individual variation or taxonomically significant morphology; however, full evaluation of these potentially diagnostic features awaits the description of multiple frontals collected from the Two Medicine Formation.

A partial skeleton of a paravian theropod (UCMP 149171) was recovered from the Kaiparowits Formation by Howard Hutchison in 1994. UCMP 149171 consists of a proximal tibia, fragmentary metatarsals, pedal phalanges, and pedal unguals, in addition to fragmentary cranial remains, including the basioccipital, fused parietals, and portions of both squamosals. Although initially considered a dromaeosaurid, several aspects of the skeleton suggest the specimen can be referred to Troodontidae, in particular the presence of a ventrally sloping cnemial crest on the tibia. Unfortunately, the specimen is extremely damaged and does not preserve enough overlapping elements to determine its relationship to *Talos* with confidence.

Finally, an isolated troodontid distal caudal vertebra and several isolated troodontid teeth are represented in the Kaiparowits materials reposited at UMNH and ALF. The distal caudal vertebra (UMNH VP uncatalogued) preserves a distinctive dorsal sulcus as in other troodontids [26]. Representative maxillary (UMNH VP 12507) and dentary (UMMH VP 11806) teeth preserve enlarged denticles. Both mesial and distal carinae of maxillary teeth are denticulate. However, mesial carinae of the maxillary teeth exhibit subtrapezoidal denticles, whereas, those on distal carinae are subtriangular. All maxillary denticles are tightly appressed and apically oriented. In contrast, denticles are restricted to the distal carinae of at least caudal dentary teeth, are pectinate in morphology (i.e., elongate and subtriangular), widely spaced, and oriented perpendicular to the long axis of the tooth.

Although the caudal vertebra, assortment of teeth (e.g., UMNH VP 12507 & UMMH VP 11806), isolated frontal (UMNH VP 16303), and associated skeleton (UCMP 149171) may ultimately prove referable to *Talos*, at present these materials do not overlap significantly with the holotype specimen. As such, there is no justification other than biogeographic and chronostratigraphic proximity for referring these materials to *Talos* and we refrain from doing so here.

### Paleobiogeographic Implications

To date, *Troodon formosus* has been reported from North American formations extending over 4000 kilometers and encompassing a temporal range of approximately 20 million years. These include the Ferris, Lance, Horseshoe Canyon, Judith River, Dinosaur Park, Hell Creek, Scollard, Two Medicine, Prince Creek, and “El Gallo” formations [16], [66]–[67]. The genus *Troodon* has also been reported from the Upper Cretaceous Kaiparowits, Wahweap, and Dakota formations of Utah and the Wapiti, St. Mary River, and Oldman formations of Alberta, Canada [64], [66], [68]. In sum, *T*. *formosus* (or at minimum *Troodon* sp.) is reported to have inhabited most of western North America throughout the Campanian and Maastrichtian epochs.

We find the likelihood that these specimens represent a single species of troodontid theropod to be low. First, many of these identifications were based solely on isolated teeth, which are not known to be autapomorphic to species level within other theropod clades [69]–[71]. Moreover, the recovery of *Talos sampsoni* indicates that not all troodontid materials from the upper Campanian of the Western Interior Basin (WIB) are referable to *Troodon formosus*. Finally, recent paleobiogeographic studies have demonstrated that even within a short window of the Campanian (e.g., 74–76 million years) coeval formations of the WIB preserve predominantly unique dinosaurian faunas [72]–[85]. These patterns are most easily recognized among megaherbivorous clades such as ceratopsids [73], [82] and hadrosaurids [80]–[81], [83], which are not only more abundantly preserved across the WIB, but also possess extensive and often bizarre species-recognition signals [86] that aid in the identification of unique taxa from fragmentary skeletal remains.

In contrast, coelurosaurian theropod specimens are comparatively conservative (i.e., they generally lack obvious species signaling structures such as horns and crests), a factor that may reflect actual lower taxonomic diversity or may act as a bias against the recognition of unique species. Conservative skeletal morphology coupled with the exceedingly fragmentary fossil record of small WIB theropods offers a potential explanation for the contemporary view that *T*. *formosus* exploited an anomalously large geographic and temporal range when compared to other, more thoroughly documented dinosaur clades of the WIB.

The hypothesis that small theropod clades of the WIB exhibited higher taxonomic diversity than currently recognized is supported by multiple lines of evidence, including the constrained paleobiogeographic ranges observed in other WIB terrestrial vertebrate clades during the late Campanian [84] and the correlation between small body-size and restricted geographic range documented in terrestrial vertebrates more generally, e.g., [87]. Although many extant carnivores, for example the mountain lion, *Felis concolor,* and coyote, *Canis latrans*, exhibit historical ranges across nearly the entirety of the Western Hemisphere and North America, respectively [88]–[89], carnivorous theropod species of the late Campanian WIB do not appear to follow the same pattern [79], [90]–[91], even among taxa of large body size (e.g., tyrannosaurids). Moreover, those theropod specimens from the Upper Cretaceous Kaiparowits Formation that have proven diagnostic all represent new species (i.e., *Hagryphus giganteus*, *Teratophoneus curriei*, and *Talos sampsoni*) and clearly demonstrate greater diversity among small theropods of the WIB than previously appreciated. In short, we hypothesize that the highly fragmentary fossil record of small theropods across the WIB together with a lack of pronounced osteological species recognition signals has led to over-synonymization of theropod taxa and that the taxonomic diversity of Late Cretaceous coelurosaurians including troodontids in North America is presently underestimated.

It is unfortunate that the holotype of *Talos* (UMNH VP 19479)—the first troodontid skeleton from the late Campanian WIB to be recognized as distinct from skeletal materials currently referred to *Troodon*—does not preserve craniodental materials that can be compared to the type specimen of *T*. *formosus*. Ultimately, a comprehensive reevaluation of troodontid materials recovered from the Late Cretaceous WIB may shed additional light on this topic; however, such an endeavor is beyond the scope of the present study.

### Hind Limb Pathology in *Talos sampsoni*

Trackway evidence indicates that the second digit of deinonychosaurians did not function as a primary weight-bearing digit during locomotion [59]. Rather, the enlarged ungual of the second digit likely functioned in prey capture as a grappling, puncturing, and/or climbing instrument [92]–[95], an intraspecific weapon analogous to the spurs of galliforms [96], or a defensive weapon as in the enlarged second pedal ungual of cassowaries [97]. It is particularly noteworthy then, that the second pedal digit on the left hind limb of *Talos* exhibits extreme pathological modification of the first phalanx (II-1). Not only is there evidence of a surface lesion on the distolateral margin of the element (Figure 13A), microCT reveals that the internal structure of the phalanx is extensively reorganized. Constraining the etiology of this type of pathology is challenging, as the generalized nature of the surface and internal bony modifications could be related to a myriad of causes, including physical trauma (e.g., fracture/puncture injury), a variety of local or systemic infectious processes, or perhaps the former followed by some variant of the latter. Moreover, any scenario for how such morphology was formed from the information at hand would be speculative at best. However, it is clear from the degree of remodeling that, at minimum, weeks to months would have transpired during which time the internal structure of the injured phalanx was reorganized and the bony collar was formed around its peripheral margin.

MicroCT data allow the reconstruction of the original and seemingly pathology-free external contour of the cortical bone. From this it is apparent that the majority of the callus is lateralized to the left side of the element, coincident with the same side as the external opening. Such a focal lesion could reflect: (1) the site at which a systemically-mediated infectious process (e.g., a non-specific hematogenous osteomyelitis) exited from the interior of the bone (i.e., it represents the cloaca associated with a suppurative osteomyelitis); (2) the site of localized bony trauma (e.g., fracture or puncture wound); or (3) the site of localized non-bony trauma (e.g., a disease process affecting the integument) that progressed to involve the phalanx. In all three cases, the bony collar or callus formation then represents secondary (i.e., post-traumatic/post-infectious) bone proliferation. Notably, such callus formation is typically associated with the reparative phase of bone healing following a fracture [98]–[100]. It also may be the case that callus formation in this specimen results not from a major (i.e., macroscopically observable) fracture, but rather, from (a) stress fracture(s) not observable in the microCT dataset. Regardless of the primary cause for the callus formation, morphology of the cloaca is consistent with suppurative osteomyelitis.

As the skeletal pathology is restricted to this single element, with perhaps concomitant minor changes in next distal element (i.e., left pedal phalanx II-2), the conservative interpretation is that this represents a localized phenomenon resulting from acute injury followed by infection and remodeling, rather than from any systemic etiology. As such, this is not entirely unexpected in a deinonychosaurian given the hypothesized use of the second pedal digit in prey acquisition/manipulation and/or conspecific aggression. Whether this morphology pertains to post-traumatic bone proliferation following a bite/scratch injury, fracture callus formation, or perhaps a combination of the two, remains a topic for future research.

Injury to the second pedal digit in *T*. *sampsoni* supports the interpretation that this digit was not only subjected to instances of potentially high loading during use, but that it was, not surprisingly, susceptible to trauma resulting directly from defensive strategies (e.g., biting, slashing) employed by prey or conspecifics. Interestingly, the proximal and distal interphalangeal joints of this digit appear to have retained at least some mobility based on the maintenance of congruent articular surfaces. It is unclear how such bone pathology may have affected performance of this digit or the entire limb with regard to both locomotor and inferred functional abilities; however, the lack of extensive remodeling across the remainder of the hind limb is consistent with the hypothesis that the hypertrophied digit II of many paravian species was not extensively employed in locomotion.

## Supporting Information

Figure S1 **Proximal histologic cross-sections of the femur of** ***Talos sampsoni*** **(UMNH VP 19479).** Medullary space is towards the bottom. Bands representing slowed growth including lamellar bone and lines of arrested growth are marked with an arrow. The proximal aspect of the section is obscured by diagenetic alteration.
(TIF)

Table S1 **Select measurements (in mm) of** ***Talos sampsoni*** **(UMNH VP 14979).** Abbreviations: CL, centrum length; CW, centrum (transverse) width; DH, distal (craniocaudal or extensor/flexor) height (*also proximodistal height of astragalar body); DW, distal (transverse) width; H, craniocaudal height; L, length; MH, midshaft (craniocaudal or extensor/flexor) height; MW, Mid-shaft (transverse) width; NS, neural spine height; PH, proximal (craniocaudal or extensor/flexor) height; PW, proximal (transverse) width. Asterisk denotes incomplete element with estimate in parentheses.
(DOC)

Dataset S1 **Phylogenetic character list, data matrix, and references for** ***Talos*** **sampsoni (UMNH VP 14979).**
(DOC)
