# Supplementary figures and images for: A New Troodontid Theropod, Talos sampsoni gen. et sp. nov., from the Upper Cretaceous Western Interior Basin of North America
Source: PLoS One. 2011 Sep 19;6(9):e24487. doi: 10.1371/journal.pone.0024487 (PMC3176273; doi:10.1371/journal.pone.0024487)

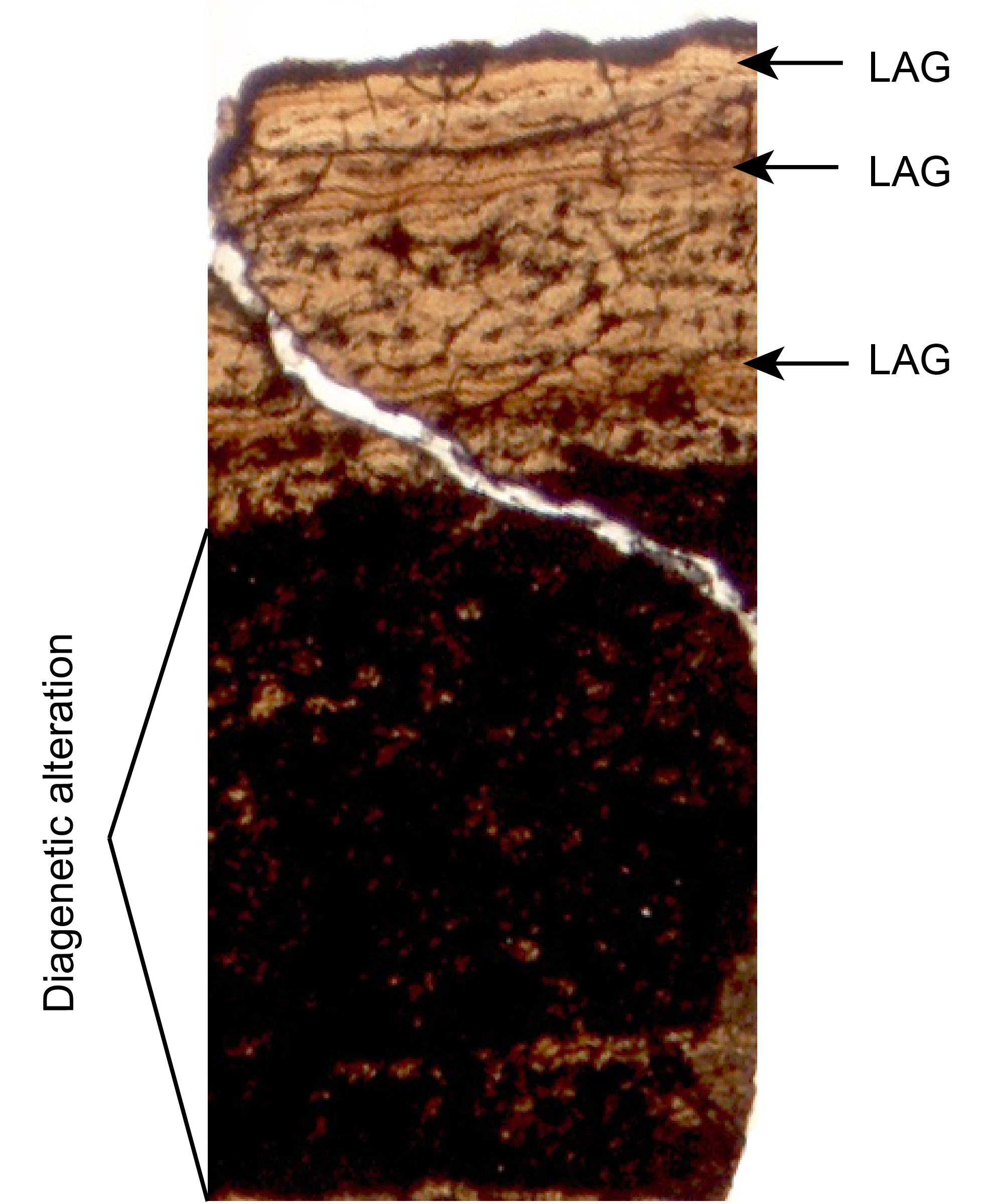

Supplement: Figure S1 — Proximal histologic cross-sections of the femur of Talos sampsoni (UMNH VP 19479). Medullary space is towards the bottom. Bands representing slowed growth including lamellar bone and lines of arrested growth are marked with an arrow. The proximal aspect of the section is obscured by diagenetic alteration. (TIF) [file pone.0024487.s001.tif]
