# Supplementary material for: A New Troodontid Theropod, Talos sampsoni gen. et sp. nov., from the Upper Cretaceous Western Interior Basin of North America
Source: PLoS One. 2011 Sep 19;6(9):e24487. doi: 10.1371/journal.pone.0024487 (PMC3176273; doi:10.1371/journal.pone.0024487)
Supplement: Table S1 — Select measurements (in mm) of Talos sampsoni (UMNH VP 14979). Abbreviations: CL, centrum length; CW, centrum (transverse) width; DH, distal (craniocaudal or extensor/flexor) height (*also proximodistal height of astragalar body); DW, distal (transverse) width; H, craniocaudal height; L, length; MH, midshaft (craniocaudal or extensor/flexor) height; MW, Mid-shaft (transverse) width; NS, neural spine height; PH, proximal (craniocaudal or extensor/flexor) height; PW, proximal (transverse) width. Asterisk denotes incomplete element with estimate in parentheses. (DOC) [file pone.0024487.s002.doc]

| ***UMNH VP 14979*** | **Measurements** | | | | | | |
| --- | --- | --- | --- | --- | --- | --- | --- |
| **Element** | *CL* | *PW* | *DH* | *DW* | *NS* | *PH* |  |
| Mid-dorsal vertebra | 22.86 | 16.26 | 23.23 | 17.71 | 29.96 | 20.69 |  |
| Sacral centrum | 19.98 | 17.92 | 13.22 | 23.15 |  | 14.7 |  |
|  | *L* | *PW* | *PH* | *MW* | *MH* | *DW* | *DH** |
| Pubic shaft | 121.91* | 6.61 | 17.32 | 7.03 | 11.08 | 12.19 | 8.61 |
| Ischium | 101.71* | 11.32* | 28.40* | 5.20 |  | 3.40 | 2.96* |
| Ulna | 93.4 | 12.25 | 10.1 |  |  | 14.01 | 6.42 |
| Tibia | 231* |  |  |  |  | 37.56 | 10.69 |
| Fibula (proximal) | 61.2* | 29.89* | 9.275* |  |  |  |  |
| Astragalus |  | 33.23 |  | 33.55 |  |  | 15.74 |
| MTII | 158.86 |  |  | 4.14 | 7.71 |  |  |
| MTIII |  |  |  |  |  |  |  |
| MTIV | 175.88 |  |  | 11.44 | 13.68 |  |  |
| PI-1 | 18.7 | 7.06 | 10.3* | 6.05 |  | 7.14 | 7.3 |
| PI-2 | 22.89 |  |  | 5.61 |  |  |  |
| PII-1 | 32.29 |  |  | 9.58 |  |  |  |
| PII-2 | 22.29 | 8.89 |  | 7.37 |  | 7.56 |  |
| PII-3 | 32.66* |  |  | 6.1 |  |  |  |
| PIII-1 | 35.58 | 14.38 | 16.13 | 8.19 |  | 13.52 | 10.63 |
| PIII-2 | 25.96 | 13.11 | 12.57 | 8.61 |  | 11.17 | 9.11 |
| PIII-3 | 25.68 | 10.52 | 11.41 | 7.19 |  | 10.2 | 9.77 |
| PIII-4 | 24.79* |  |  | 6.75 |  |  |  |
| PIV-1 | 22.64* | 11.12 | 14.22 | 9.1 |  | 11.55 | 10.7* |
| PIV-2 | 21.11* | 10.5 | 12.99 | 8.72 |  | 10.21 | 9.74 |
| PIV-3 | 17.12 | 9.49 | 10.76 | 8.3 |  | 8.79 | 8.66 |
| PIV-4 | 18.02 | 8.08 | 9.8* | 6.44 |  | 7.97 | 8.38 |
| PIV-5 | 18.45 |  |  | 5.76 |  |  |  |
