# Supplementary material for: A New Troodontid Theropod, Talos sampsoni gen. et sp. nov., from the Upper Cretaceous Western Interior Basin of North America
Source: PLoS One. 2011 Sep 19;6(9):e24487. doi: 10.1371/journal.pone.0024487 (PMC3176273; doi:10.1371/journal.pone.0024487)
Supplement: Dataset S1 — Phylogenetic character list, data matrix, and references for Talos sampsoni (UMNH VP 14979). (DOC) [file pone.0024487.s003.doc]

**Dataset S1. Phylogenetic character list, data matrix, and references**

**Character list**

Characters 1-38 from Norell et al. (2000). Character state changes: character 2, IGM/100/44 changed to state (0); character 12, *Byronosaurus, Zanabazar, Saurornithoides* changed to state (0); characters 32 and 34 descriptions and states modified as listed.

1. Teeth few (<100) (0), or numerous (1).

2. Absence (0) or presence (1) of enlarged, distally oriented denticles.

3. Teeth serrated (0) or unserrated (1).

4. Osseous canal for maxillary branch of CN V absent (0) or present (1).

5. Maxilla excluded from narial border (0) or participating in narial border (1).

6. Accessory antorbital fenestra small (0) or enlarged (1).

7. Dentary labial foramina situated outside of groove (0) or in a horizontal groove (1).

8. Inflated para-basisphenoid absent (0) or present (1).

9. Lacrimal L-shaped (0) or T-shaped (1).

10. Prefrontal absent (0) or present (1).

11. Anterior ramus of lacrimal short (0) or long (1).

12. Trigeminal nerve exiting through one opening (0) or two or more openings (1).

13. Metotic fissure open (0) or constricted (1).

14. Foramen magnum subcircular or wider than tall (0) or taller than wide (1).

15. Basisphenoidal recess absent (0) or present (1).

16. Basal tubera shallow (0) or deep (1).

17. Notch between basal tubera large and U-shaped (0) or small, V-shaped incisure (1).

18. Paroccipital processes elongate and squared-off (0) or abbreviated (1).

19. Posterior tympanic recess invades body of paroccipital process (0) or paroccipital closed off

(1).

20. Enlarged subotic recess absent (0) or present (1).

21. Quadrate non-pneumatic (0) or pneumatic (1).

22. Anterior tympanic recess consists of separate prootic recess and basisphenoid recess (0) or

prootic recess confluent with subotic and delimited by enlarged otosphenoidal crest

(lateral depression) (1).

23. Dorsal and ventral dentary margins subparallel (0) or distally convergent (1).

24. Dentaries straight in ventral view (0) or with slight medial curvature at symphyseal extremity

(1).

25. Splenial concealed in lateral view (0) or with large lateral exposure (1).

26. Large axial epipophyses absent (0) or present (1).

27. Carotid processes absent (0) or present on posterior cervical vertebrae (1).

28. Large hypapophyses on anterior dorsals absent (0) or present (1).

29. Tip of dorsal neural spines unexpanded (0) or expanded to form spine table (1).

30. Distal caudals with low neural spine or ridge (0) or with sagittal sulcus above neural canal

(1).

31. Proximal gastralia shorter than distal gastralia (0) or proximal segments longer (1).

32. Obturator process positioned distally (0) or at midshaft (1). (modified from Norell et al.

(2000), coding changed based on Norell et al. (2009))

33. Greater and lesser trochanters widely separated by deep notch (0) or appressed or fused

together (1).

34. Rugose lateral ridge on proximal part of femoral shaft absent (0) or present (1).

35. Third metatarsal only slightly constricted (0) or strongly constricted and concealed from

anterior view (1). (modified from Norell et al. (2000), coding of *Mei* based on Xu and

Norell (2004)).

36. Metapodium symmetrical with mt II equal to mt IV in length (0) or mt II slender and shorter

than mt IV (1).

37. Pedal phalanx II-2 elongate and without proximal heel (0) or abbreviated and tall with

proximal heel (1).

38. Enlarged raptorial claw on digit II absent (0) or present (1).

39. Pelvis opisthopubic (0) or vertical to propubic (1).

40. Caudodorsal ridge on ischium absent (0) or present originates near iliac peduncle

(1) or originates further distally from iliac peduncle (2).

41. Groove delineating base of ascending process of astragalus craniocaudally narrow (0) or

hypertrophied, craniocaudally wide (1).

42. Bony ‘tab’ on dorsolateral aspect of third metatarsal proximal to distal articular condyles

poorly developed to absent (0) or pronounced (1).

43. Fourth metatarsal with gentle lateral curve (0) or with kink in shaft (1).

44. Fourth metatarsal without (0) or with notch in dorsolateral aspect of distal condyle (1).

**Data matrix**

Dromaeosauridae

00000000110000100010000011?1111(0&1)110011(0&1)00000

Avialae

0-1100000???1?????100?000?1(0&1)00101?000(0&1)000000

Mei long

?-1?1?1???????????101?10?0?101?0000111??0?0?

Sinovenator changi

?00?0110?????1?010?01010?0?0???01001??000000

IGM 100/44

100???1?????0?101??01????0????????1111???10?

Sinornithoides youngi

100?111??11???0???????101011?010111111??0?0?

Byronosaurus jaffei

1-111111111011001111?11011?1?1??1???1???????

Zanabazar junior

110111111110?1001111??111????1????11????????

Saurornithoides mongoliensis

1101111??-?0??0?11?11?111??????111111111??1?

Troodon formosus

110?11111111110111011111111111?11(0&1)1111121011

Talos sampsoni

?????????????????????????????1?1?11111?11111

**References**

Norell MA, Makovicky PJ, Clark JM (2000) A new troodontid theropod from Ukaah Tolgod, Mongolia. J Vertebr Paleontol 20: 7–11.
